# LPS Unmasking of *Shigella flexneri* Reveals Preferential Localisation of Tagged Outer Membrane Protease IcsP to Septa and New Poles

**DOI:** 10.1371/journal.pone.0070508

**Published:** 2013-07-25

**Authors:** Elizabeth Ngoc Hoa Tran, Matthew Thomas Doyle, Renato Morona

**Affiliations:** Discipline of Microbiology and Immunology, School of Molecular and Biomedical Science, University of Adelaide, Adelaide, Australia; Universidad Nacional de La Plata, Argentina

## Abstract

The *Shigella flexneri* outer membrane (OM) protease IcsP (SopA) is a member of the enterobacterial Omptin family of proteases which cleaves the polarly localised OM protein IcsA that is essential for *Shigella* virulence. Unlike IcsA however, the specific localisation of IcsP on the cell surface is unknown. To determine the distribution of IcsP, a haemagglutinin (HA) epitope was inserted into the non-essential IcsP OM loop 5 using Splicing by Overlap Extension (SOE) PCR, and IcsP^HA^ was characterised. Quantum Dot (QD) immunofluorescence (IF) surface labelling of IcsP^HA^ was then undertaken. Quantitative fluorescence analysis of *S. flexneri* 2a 2457T treated with and without tunicaymcin to deplete lipopolysaccharide (LPS) O antigen (Oag) showed that IcsP^HA^ was asymmetrically distributed on the surface of septating and non-septating cells, and that this distribution was masked by LPS Oag in untreated cells. Double QD IF labelling of IcsP^HA^ and IcsA showed that IcsP^HA^ preferentially localised to the new pole of non-septating cells and to the septum of septating cells. The localisation of IcsP^HA^ in a rough LPS *S. flexneri* 2457T strain (with no Oag) was also investigated and a similar distribution of IcsP^HA^ was observed. Complementation of the rough LPS strain with *rmlD* resulted in restored LPS Oag chain expression and loss of IcsP^HA^ detection, providing further support for LPS Oag masking of surface proteins. Our data presents for the first time the distribution for the Omptin OM protease IcsP, relative to IcsA, and the effect of LPS Oag masking on its detection.

## Introduction

*Shigella flexneri* is an intracellular pathogen which causes bacillary dysentery, a disease characterised by the presence of severe mucoid bloody diarrhoea and by invasion of the gut epithelium [1], [2]. IcsA (VirG) is a 120 kDa outer membrane (OM) protein localised at the cell pole [3]. It mediates intracellular cytoplasmic movement of *S. flexneri* in epithelial cells, and cell-to-cell spread, by the assembly of an F-actin comet-tail at one pole of the bacterium [4]–[6]. This type of movement is described as actin-based motility (ABM). IcsA is secreted primarily at the ‘old pole’ of Shigellae [7] which is opposite the ‘new pole’ (the pole derived from the site of septation of the parent cell [8]. The 36.9 kDa IcsP (SopA) OM protease of *S. flexneri* slowly cleaves IcsA at the Arg_758_– Arg_759_ bond position [9] resulting in the release of a 95 kDa amino-terminal IcsA fragment that can be detected in culture supernatants [5], [10]. Analysis of *icsP*/*sopA* mutants has shown that IcsA is detected across the entire surface of these bacteria with polar reinforcement [11], [12]. Over-expression of IcsP results in the complete removal of IcsA from the cell surface [13].

IcsP belongs to the Omptin family of proteases which consists of 6 members; OmpT and OmpP of *Escherichia coli*, Pla of *Yersinia pestis*, PgtE of *Salmonella enterica*, Pla endopeptidase A of *Erwinia pyrifoliae*, and IcsP of *Shigella flexneri*. Immunogold labelling of overexpressed OmpP has shown that OmpP is symmetrically distributed over the cell surface [14]. However to date, no studies have attempted to describe the surface localisation of Omptins expressed at native levels. While it has been suggested that IcsP may also be located uniformly across the cell surface [13], its specific distribution is currently unknown. In contrast to many inner membrane proteins, such as FtsZ [15] and MreB [16] involved in cell division and cell shape, few OM proteins have had their subcellular distribution determined. An exception to this is the *E. coli* OM protein LamB which has been characterised to exist as two populations: one that diffuses in a helical pattern, and one that is relatively immobile [17], [18]. The *E. coli* Iss and Bor proteins have been detected on the cell surface with no distinct pattern [19]. A number of non-specific *E. coli* OM proteins were suggested to be organised in stable helical swaths [20], and data by Shiomi *et al*. (2006) suggested that the general protein translocation Sec machinery itself may also be arranged in a helical array. Whether IcsP possesses a distribution similar to these OM proteins, or has an asymmetric distribution like IcsA, is the subject of this study.

In addition to the above, mutations affecting lipopolysaccharide (LPS) have also been shown to affect the observed distribution of OM proteins [21]–[24]. LPS is composed of three distinct regions: lipid A, core sugars and O antigen (Oag) polysaccharide chains. Strains with LPS containing all 3 regions intact are known as smooth LPS strains. *Shigella* mutants lacking Oag are known as rough LPS strains. Such strains have been shown to have high levels of circumferentially distributed IcsA on the cell surface (at both cell poles and on lateral regions) [25], [26], compared to the polar localisation of IcsA seen in smooth LPS strains. Treatment of Y serotype derivatives of smooth LPS *S. flexneri* with bacteriophage Sf6 tailspike protein (TSP) endorhamnosidase results in the hydrolysis of Oag chains and an increase detection of circumferential IcsA on the cell surface by indirect immunofluorescence (IF) staining [21]. This suggests that the presence of LPS Oag masks the observed distribution of IcsA on the cell surface and supports the idea that LPS Oag structure may block antibody accessibility to the detection of surface proteins [22], [23]. The effect of LPS Oag structure on the detection and distribution of IcsP has not been investigated.

In this study, we investigated the distribution of IcsP by cell surface quantum dot (QD) IF labelling of functional, HA-tagged IcsP (IcsP^HA^) in *S. flexneri* 2457T and establish that LPS Oag masks detection of IcsP^HA^ on the cell surface by using tunicamycin to inhibit Oag synthesis. Additional IF labelling with anti-IcsA antibodies to mark the location of the old pole suggested that IcsP is preferentially localised to the new pole of non-septating cells and to the septa of septating cells. We also investigated the distribution of IcsP in a rough LPS 2457T strain to provide further support for the LPS Oag masking hypothesis. Overall, our data presents for the first time the cell surface distribution of the Omptin OM protease IcsP and the effect of LPS Oag masking on its detection. This distribution has implications for IcsA polarity determination, and a model is described to explain IcsP’s contribution to IcsA polarity in *S. flexneri*.

## Methods

### Ethics Statement

The anti-IcsP and anti-IcsA antibodies were produced under the National Health and Medical Research Council (NHMRC) Australian Code of Practice for the Care and Use of Animals for Scientific Purposes and were approved by the University of Adelaide Animal Ethics Committee.

### Bacterial Strains, Plasmids and Media

Bacterial strains and plasmids used in this study are listed in Table 1. Bacteria were routinely grown at 37°C in Luria-Bertani (LB) broth with aeration or on Congo red agar [26]. Antibiotics used were as follows: 50 µg ampicillin (Amp) ml^−1^; 25 µg chloramphenicol (Cml) ml^−1^; 50 µg kanamycin (Kan) ml^−1^.

**Table 1:** Bacterial strains and plasmids.

Strain or plasmid	Relevant characteristics	LPS*	Source/reference
***E. coli*** ** K-12**			
DH5α	*endA hsdR supE44 thi-1 recA1 gyrA relA* Δ(*lacZYA-argF*) U169 [φ80d*lac*Δ(*lac*Z) M15)	R	Gibco-BRL
***S. flexneri*** ** 2a**			
2457T	wild type strain	S	[26]
ETRM22	2457T *icsP^-^* mutant; Kan^R^	S	This study
ETRM230	2457T *rmlD::kan^R^* mutant; Kan^R^	R	This study
ETRM233	2457T *rmlD^-^* mutant; Kan^R^	R	This study
ETRM240	2457T *icsP^−^/rmlD^-^* mutant; Kan^R^	R	This study
ETRM143	ETRM22 (pIcsP)	S	This study
ETRM117	ETRM22 (pIcsP^HA^)	S	This study
ETRM118	ETRM22 (pBAD30)	S	This study
ETRM243	ETRM240 (pIcsP^HA^)	R	This study
ETRM245	ETRM240 (pBAD30)	R	This study
RMA4376	ETRM243 (pRMA727)	S	This study
RMA4377	ETRM243 (pACYC184)	S	This study
**Plasmids**			
pCACTUS	Suicide vector; Cml^R^; 30°C		[27]
pCACTUS-*icsP*::*kan^R^*	pCACTUS with *icsP::kan^R^* gene		This study
pSL1180	Cloning vector; Amp^R^		[46]
pSL1180-*icsP*::*kan^R^*	pSL1180 with *icsP::kan^R^* gene		This study
pACYC184	Cloning vector; Cml^R^, Tet^R^		[47]
pKTUWE	pACYC184 with *kan^R^* gene; Kan^R^		[36]
pKD4	Vector containing FRT-flanked *kan^R^* gene		[32]
pKD46	Red lambda plasmid; Amp^R^; 30°C		[32]
pCP20	FLP recombinase; Amp^R^, Cml^R^; 30°C		[32]
pGEMT-Easy	Cloning vector; Amp^R^		Promega
pGEMT-Easy::*icsP* ^HA^	pGEMT-Easy with *icsP^HA^* gene; Amp^R^		This study
pGEMT-Easy::*kan^R^*	pGEMT-Easy with *kan^R^* gene; Amp^R^		This study
pBAD30	Arabinose-inducible pBAD promoter vector, Amp^R^		[39]
pIcsP	pBAD30 with *icsP* gene; Amp^R^		This study
pIcsP^HA^	pBAD30 with *icsP^HA^* gene; Amp^R^		This study
pRMA718	pUC1318 containing *S. flexneri rfb* region; Amp^R^		[26]
pRMA727	pACYC184 with *rmlD* gene; Cml^R^		[26]

* S, smooth LPS; R, rough LPS.

### DNA Methods

*E. coli* K-12 DH5α was used for all cloning experiments. DNA manipulation, PCR, transformation and electroporation was performed as previously described [27], [28]. Anti-HA monoclonal antibody (#H3663) was purchased from Sigma. Rabbit anti-IcsP and anti-IcsA antibodies were prepared as described previously [26], [29]. The antibodies were produced under the National Health and Medical Research Council (NHMRC) Australian Code of Practice for the Care and Use of Animals for Scientific Purposes and were approved by the University of Adelaide Animal Ethics Committee.

### Insertion of HA Epitope into IcsP

The sequence encoding the HA epitope (YPYDVPDYA) was inserted into the putatively non-essential IcsP OM loop 5 (based on sequence alignments with *E. coli* OmpT) using SOE PCR [30], [31]. In the first part of this two-step PCR technique, upstream and downstream amplicons were amplified from *S. flexneri* 2457T genomic DNA using HA encoding primers (ET18/ET19) and *icsP* ^-^ specific primers (ET3/ET10) (Table 2). The two amplicons from this primary PCR were then mixed and used as a DNA template for the second round of PCR with primers ET3/ET10. In this second reaction, the HA encoding regions of the primary PCR amplicons overlap and prime one another to give the final *icsP* PCR product with an inserted HA epitope. The *icsP^HA^* fragment was then cloned into pGEMT-Easy and primers ET22/ET25 (Table 2) were used to amplify an *icsP* ^HA^ product with *Kpn*I and *Hind*III restriction enzyme sites from this construct. The resultant *Kpn*I-*Hind*III fragment was digested and sub-cloned into likewise digested pBAD30 to give pBAD30::*icsP* ^HA^, also referred to as pIcsP^HA^ in text (Table 1). Primers ET22/ET25 were also used to amplify the *icsP* gene from 2457T genomic DNA, and cloned into pBAD30 to give pBAD30::*icsP*, referred to as pIcsP in text (Table 1). DNA sequencing was used to confirm that no mutation had been introduced by PCR into the sequence, and the presence of the in-frame HA epitope tag sequence.

**Table 2:** Oligonucleotides used in this study.

Primer name	Oligonucleotide sequence (5′–3′)*	Target	Genebank	nt position†
*HA-encoding primers*				
ET18	tacccgtacgacgtcccggactacgccagtaccaatatatctggcac	*icsP* gene	AF386526	221158
ET19	ggcgtagtccgggacgtcgtacgggtattgctcataaagagatgtatc	*icsP* gene	AF386527	221157
*icsP-specific primers*				
ET3	gcggatccgtattgcttctgccatttcc	484 bp upstream *icsP*	AF386526	219783
ET4	gcgagctcgtccctgatagcactgttc	371 bp downstream *icsP*	AF386526	221618
ET10	gcggatccaaaaatatactttatacctgcg	*icsP* gene	AF386526	221223
ET22	gcggtaccataaagtaagaagatcatggac	16 bp upstream *icsP*	AF386526	220251
ET25	gggaagctttcaaaaaatatactttatacctg	*icsP* gene	AF386526	221225
*pKD4 specific primers*				
ET28	ccgggctagctgtgtaggctggagctgcttcg	FRT-*kan^R^* priming site 1	AY048743	31
ET29	gcccgctagccatatgaatatcctcctta	FRT-*kan^R^* priming site 2	AY048743	1488

* Underlined sequences indicate the nucleotides that encode the HA epitope.

† nt, nucleotide.

### Construction of *S. flexneri icsP* mutant

The *S. flexneri* 2457T *icsP^-^* mutant strain was constructed using allelic exchange mutagenesis [27] to inactivate the *icsP* gene by insertion of a kanamycin resistance gene (*kan^R^*). Initially, the *icsP* gene was PCR amplified with primers ET3/ET4 containing *Bam*HI and *Sac*I restriction sites from 2457T genomic DNA. The resultant PCR fragment was digested with *Bam*HI and *Sac*I and sub-cloned into likewise digested pSL1180 (Table 1). Further digestion with *Cla*I allowed insertion of the *Acc*I-*Acc*I digested *kan^R^* gene from pKTUWE (Table 1) to give pSL1180-*icsP*::*kan^R^* (Table 1). Following re-digestion with *Bam*HI and *Sac*I, the *icsP*::*kan^R^* fragment was cloned into pCACTUS (Table 1) and transformed into *S. flexneri* 2457T via electroporation. Allelic exchange mutagenesis was performed as previously described [27]. The *icsP*::*kan^R^* mutation in the virulence plasmid was confirmed by PCR with primers ET3/ET4 (Table 2) to give the 2457T *icsP^-^* mutant ETRM22 (Table 1).

### Construction of *S. flexneri icsP^−^/rmlD^-^* Double Mutant

The *S. flexneri* 2457T *icsP^−^/rmlD^-^* mutant strain was constructed using a modification of the λ Red recombinase system to initially delete the *rmlD* gene [32]. Primers ET28/ET29 containing *Nhe*I restriction enzyme sites (Table 2) were used to PCR amplify the *kan^R^* gene from pKD4 (Table 1). The amplified product was ligated into pGEMT-Easy and pGEMT-Easy::*kan^R^* was digested with *Nhe*I. The *Nhe*I-*kan^R^-Nhe*I fragment was then subcloned into likewise digested pRMA718 [26] to give pRMA718-*rmlD*::*kan^R^* (Table 1). This plasmid was then digested with *Bam*HI and the *rmlD::Kan^R^* fragment was cloned into the *Bam*HI site of pCACTUS. The pCACTUS-*rmlD::Kan^R^* construct was then electroporated into *S. flexneri* 2457T and allelic exchange mutagenesis was induced to give the 2457T *rmlD*::*kan^R^* mutant ETRM230 (Table 1). ETRM230 was transformed with pCP20 at 30°C to flip out the FRT flanked *kan^R^* gene and give the 2457T *rmlD^-^* mutant ETRM233 (Table 1). The *rmlD* mutation was confirmed by LPS analysis. ETRM233 was further electroporated with pCACTUS-*icsP::kan^R^* (Table 1) and another round of allelic exchange mutagenesis was performed to give the final 2457T *rmlD^−/^icsP^-^* double mutant ETRM240 (Table 1).

### Analysis of IcsP/IcsP^HA^ Protein Production

For detection of native IcsP, strains were grown at 37°C in LB broth with aeration for 16 h, subcultured 1/20 into fresh broth and grown for another 3 h to an OD_600_ of ∼1. Strains harbouring pIcsP or pIcsP^HA^ were grown in LB broth containing 0.2% (w/v) glucose for 16 h with aeration, subcultured 1/20 into fresh broth and grown for 1.5 h to an OD_600_ reading of ∼0.4. Cultures were then pelleted by centrifugation (2219×*g*, 10 min, Sigma 3K15 centrifuge), washed 3 times in LB, and unless otherwise stated, induced with 0.03% (w/v) arabinose for 1 h to an OD_600_ of ∼1. Cells (5×10^8^) were then harvested by centrifugation and resuspended in 2X sample buffer [33]. Protein samples were solubilised at 100°C for 5 min, separated on SDS 15% polyacrylamide gels, and stained with Coomassie R-250, or subjected to Western immunoblotting on nitrocellulose membrane (Medos) with either polyclonal rabbit anti-IcsP antiserum (at 1/250 dilution) or monoclonal mouse anti-HA (at 1/500 dilution). Detection was performed with goat anti-rabbit (or anti-mouse) horseradish-peroxidase-conjugated antibodies (KPL) and chemiluminescence reagent (Sigma). Benchmark prestained molecular weight markers (Invitrogen) were used as molecular size markers.

### Sucrose Gradient Density Fractionation

Fractionation of the cell whole membrane (WM) into cytoplasmic membrane (CM) and OM fractions was performed by sucrose gradient centrifugation according to the method of Osborn and Munson [34]. In brief, 200 ml cultures were grown and induced with arabinose as described above, harvested by centrifugation (9,800×*g*, 15 min, 4°C, JA14 rotor, Beckman centrifuge J2-21M), washed in 50 mM Tris-HCl (pH 8.0) and resuspended in 5 ml 10 mM HEPES in 1 mM MgCl_2_. The bacterial suspension was then passed through a pre-cooled French Pressure cell (SLM Aminco) once and re-centrifuged to remove cell debri. WM pellets were collected by ultracentrifugation (115,000×*g*, 1 h, 4°C, 80 Ti rotor, Beckman Coulter Optima L-100 XP ultracentrifuge), solubilised in 0.8 ml 25% (w/w) sucrose in 5 mM EDTA and applied to a 10 ml sucrose gradient of 30–50% (w/w) sucrose in 5 mM EDTA. Centrifugation to equilibrium was performed with a Beckman SW40Ti swing out rotor (217,000×*g*, 20 h, 4°C, Beckman Coulter Optima L-100 XP ultracentrifuge) and 0.5 ml fractions collected through the pierced bottom of the tube. 10 µl samples of each fraction were resuspended in 2X sample buffer [33] and IcsP protein detected as described above.

### Detection of Cell Associated and Soluble IcsA

Whole cell and supernatant bacterial protein extracts were prepared as described previously [35]. IcsA protein was detected from 10 µl whole cell protein extracts and 20 µl supernatant protein extracts. Western immunoblotting was performed as described above, but with a polyclonal rabbit IcsA antibody and a goat anti-rabbit HRP conjugate.

### LPS Analysis

LPS samples and gels were prepared as described previously [36], [37].

### LPS Depletion-regeneration Assay

Depletion and regeneration of LPS was performed as previously described [29] with the exception that 0.03% (w/v) arabinose induction was included in the final hour of tunicamycin/polymyxin B nonapeptide (PMBN) treatment.

### Formaldehyde Fixation of Bacteria for Immunofluorescence (IF) Microscopy

Bacteria were grown and induced as described above 1×10^8^ cells of induced bacteria were then harvested by centrifugation, washed once in PBS and resuspended in 100 µl 3.7% (w/v) formaldehyde (Sigma) in PBS for 20 min at room temperature (RT). Fixed bacteria were then pelleted, washed three times in PBS and resuspended in a final volume of 100 µl PBS.

### Quantum Dot (QD) IF Staining and Epi-fluorescence Microscopy

Sterile glass coverslips were placed into wells of a 24-well tray and coated with 10% (v/v) poly-L-lysine solution (Sigma) in PBS for 1 h at RT. Coating solution was aspirated and 5 µL of formaldehyde fixed bacteria were spotted onto coverslips. The tray was then centrifuged to assist adherence of bacteria (Heraeus Labofuge 400R Centrifuge, 2,000×*g*, 5 min, 20°C). Bacteria were blocked for 1 h at RT with 10% (v/v) foetal calf serum (FCS) diluted in PBS. For labelling, bacteria were incubated for 2 h at RT with mouse anti-HA antibody (Sigma) and rabbit anti-IcsA antiserum diluted 1∶50 and 1∶100 respectively in PBS containing 10% (v/v) FCS. Bacteria were then washed 3 times with PBS, and then incubated for 1 h at RT with either QD 525 donkey anti-mouse antibody (Invitrogen) or QD 625 donkey anti-rabbit antibody (Invitrogen) diluted 1∶50 and 1∶100, respectively, in PBS containing 10% (v/v) FCS. After a final 3 more washes with PBS, coverslips were mounted on glass microscope slides with Mowiol 4–88 (Calbiochem). All microscopy images were captured using an Olympus IX-7- Microscope, with a phase contrast 100X oil immersion objective and a 1.5X enlarger, which was controlled by MetaMorph (Version 7.7.1.0, Molecular Devices). All IcsP^HA^ (525 nm) and IcsA (625 nm) channel images were acquired with 1 sec and 0.1 sec exposures respectively using an X-Cite 120Q lamp set at high intensity as the excitation source. The excitation filter used was FF01-435/40-25 (Semrock) and the emission filters were FF01-525/15-25 and FF01-625/15-25 (Semrock). Semrock FF510-Di01-25×36 dichroics were used.

### Fluorescence Quantification of QD Labelled Surface IcsP^HA^

Data for intensity profile plots were extracted from images using MetaMorph’s Line-scan function which averages intensities across the perpendicular axis of a point-to-point scan. Single scans were conducted from pole-to-pole with width (perpendicular axis) equal to the bacterium (approx. 20 pixels). Cumulative scans of the 525 nm wavelength (IcsP^HA^) for non-septating cells were conducted for 50 bacteria from each independent sample. Each bacterium was scanned from the new pole to old pole where IcsA was used as a marker of the old pole. From the same samples, all septating cells from captured images were scanned starting from the septum to the old pole of one daughter cell chosen at random. An average of 26.8 septating bacteria were scanned for each sample. Intensity data was then exported to MS Excel using MetaMorph’s Dynamic Data Exchange and subsequently analysed using GraphPad Prism. Statistical significances were tested by Student’s two-tailed t-test.

## Results

### Detection of IcsP Expression by Western Immunoblotting and Immunofluorescence

**Figure 1:**
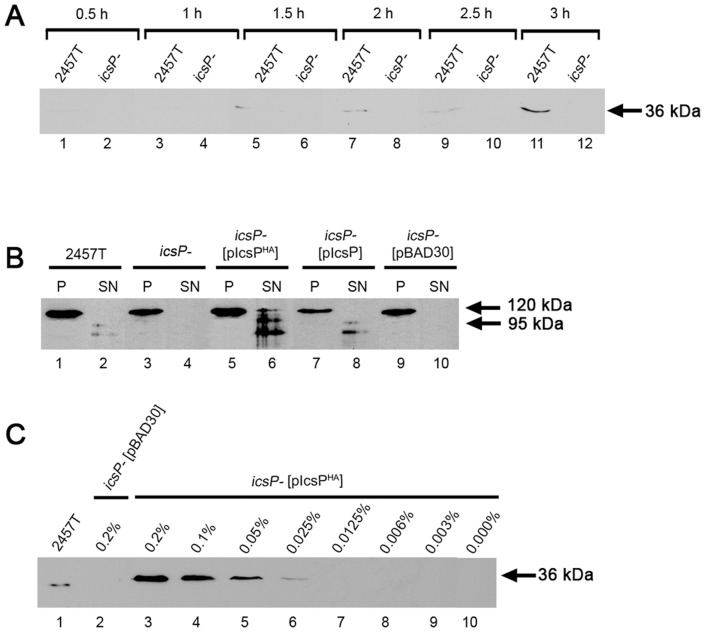
Detection of IcsP/IcsP^HA^ expression and activity on IcsA by Western immunoblotting. (**A**) *S. flexneri* strains 2457T and 2457T *icsP^-^* mutant (*icsP^-^*) were grown in LB and whole cell lysate samples were taken at 0.5, 1, 1.5, 2, 2.5 and 3 h after subculture, followed by electrophoresis on a SDS 15 % polyacrylamide gel and Western immunoblotting with rabbit anti-IcsP antiserum; (**B**) *S. flexneri* strains 2457T, *icsP^-^* and *icsP^-^* harbouring pIcsP, pIcsP^HA^ or pBAD30 (as indicated) were grown in LB for 1.5 h to an OD600 reading of ∼0.4, washed 3 times, and induced with arabinose for 1 h. Pellet and supernatant protein samples were then prepared and electrophoresed on a SDS 15 % polyacrylamide gel, followed by Western immunoblotting with rabbit anti-IcsA antibodies. The size of the full length IcsA protein (120 kDa) and the cleaved form of IcsA (95 kDa) are indicated; (**C**) *S. flexneri* strains 2457T and *icsP^-^* harbouring pIcsP^HA^ or pBAD30 were grown in LB as described in (B), followed by induction with 0%, 0.003%, 0.006%, 0.0125%, 0.025%, 0.05%, 0.1% or 0.2% (w/v) arabinose for 1 h. Whole cell lysates were prepared and electrophoresed on a SDS 15 % polyacrylamide gel, followed by Western immunoblotting with rabbit anti-IcsP antiserum. The size of the full length IcsP protein (36 kDa) is indicated in (A) and (C). Each lane contains 5 x 10^7^ bacterial cells of each strain.

In initial experiments to detect cell surface IcsP, the optimal time point for IcsP expression in *S. flexneri* was determined. Western immunoblotting with a rabbit anti-IcsP was performed on whole cell lysates collected from wild-type *S. flexneri* 2457T and the 2457T *icsP* ^-^ mutant (*icsP* ^-^) grown for 0.5, 1, 1.5, 2, 2.5 and 3 h after subculture. A band consistent with the size of the IcsP protein (∼36 kDa) was detected at time points after 1.5 h for 2457T (Fig. 1A, lanes 5, 7, 9 & 11) with high expression observed at 3 h (Fig. 1A, lane 11). We reasoned that IcsP levels at this time point were high enough for subsequent immunofluorescent detection of IcsP. No expression of IcsP was observed for *icsP* ^-^ as expected (Fig. 1A, lanes 2, 4, 6, 8, 10 & 12). Subsequent attempts to detect IcsP on the surface of 2457T with rabbit anti-IcsP however were unsuccessful, even in a rough LPS strain (data not shown). We speculate that as the IcsP protein used to raise antisera was purified under denaturing conditions, this may have affected the resulting antibody’s ability to detect native IcsP. However, difficulties with IF detection of cell surface IcsP with a polyclonal antibody have also been reported by others [13]. Hence, in an alternative approach to investigate the distribution of IcsP on the bacterial cell surface, a HA epitope was inserted into the IcsP protein.

### Insertion of a HA Epitope into IcsP

**Figure 2:**
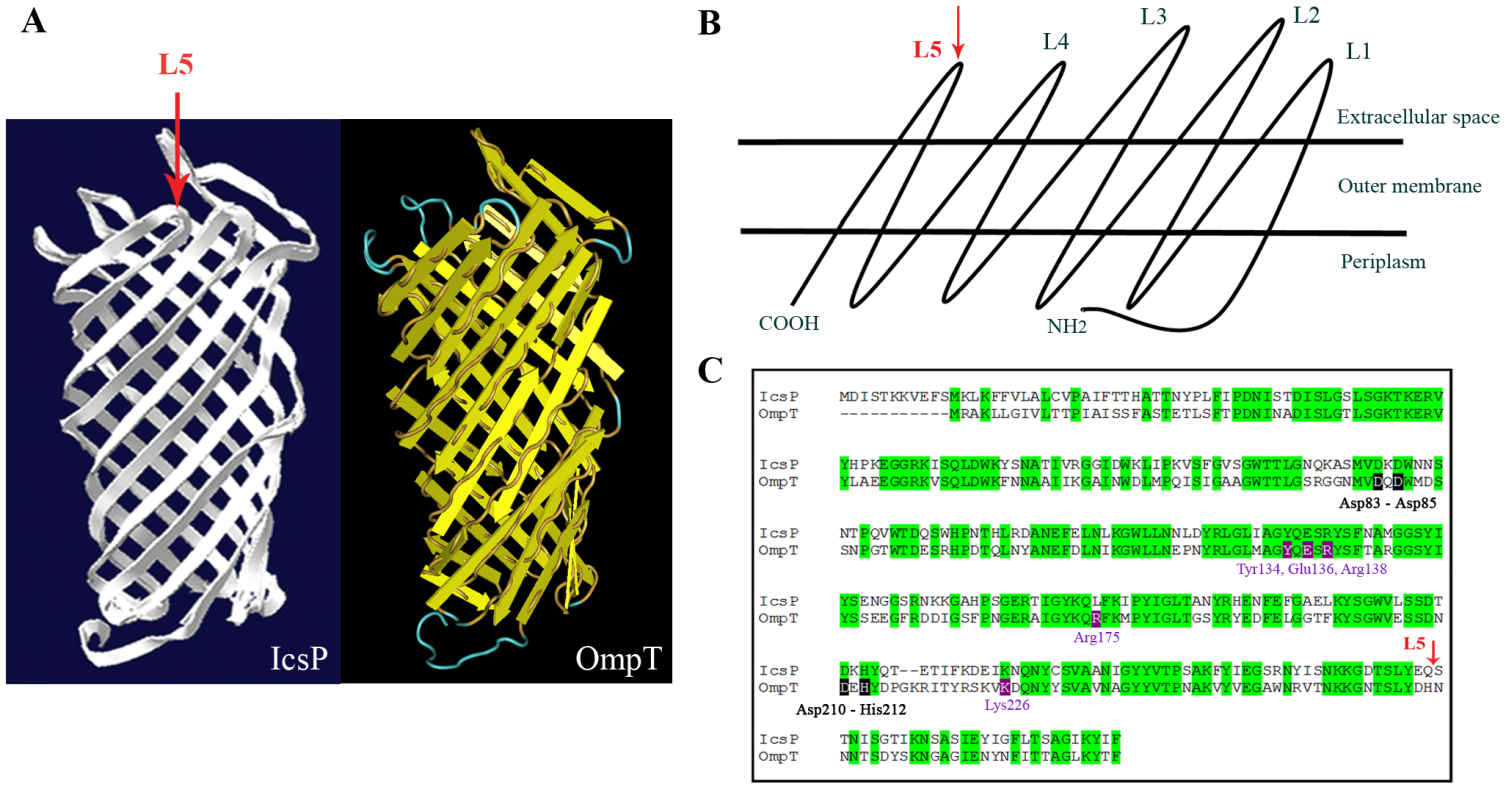
Putative structure of IcsP and location of HA epitope insertion in IcsP. (**A**) IcsP was modelled using the SWISS-MODEL Protein Modelling Server (http://swissmodel.expasy.org//SWISS-MODEL.html) (left) and compared to the structure of OmpT (PDB 1I78) (right); (**B**) Schematic diagram of IcsP showing the location of the OM loops 1 - 5; (**C**) Amino acid sequence alignment of IcsP (AF001633) and OmpT (P09169) showing 60% identity (green shaded regions). The black-boxed amino acids in the sequence of OmpT refer to active site residues found in OM loops 2 and 4 38], and the purple-boxed amino acids refer putative LPS binding sites 38]. The location of the HA epitope (YPY DVP DYA) insertion into the putatively non-active OM loop 5 (L5) is indicated by the red arrow in (A) – (C).

**Figure 3:**
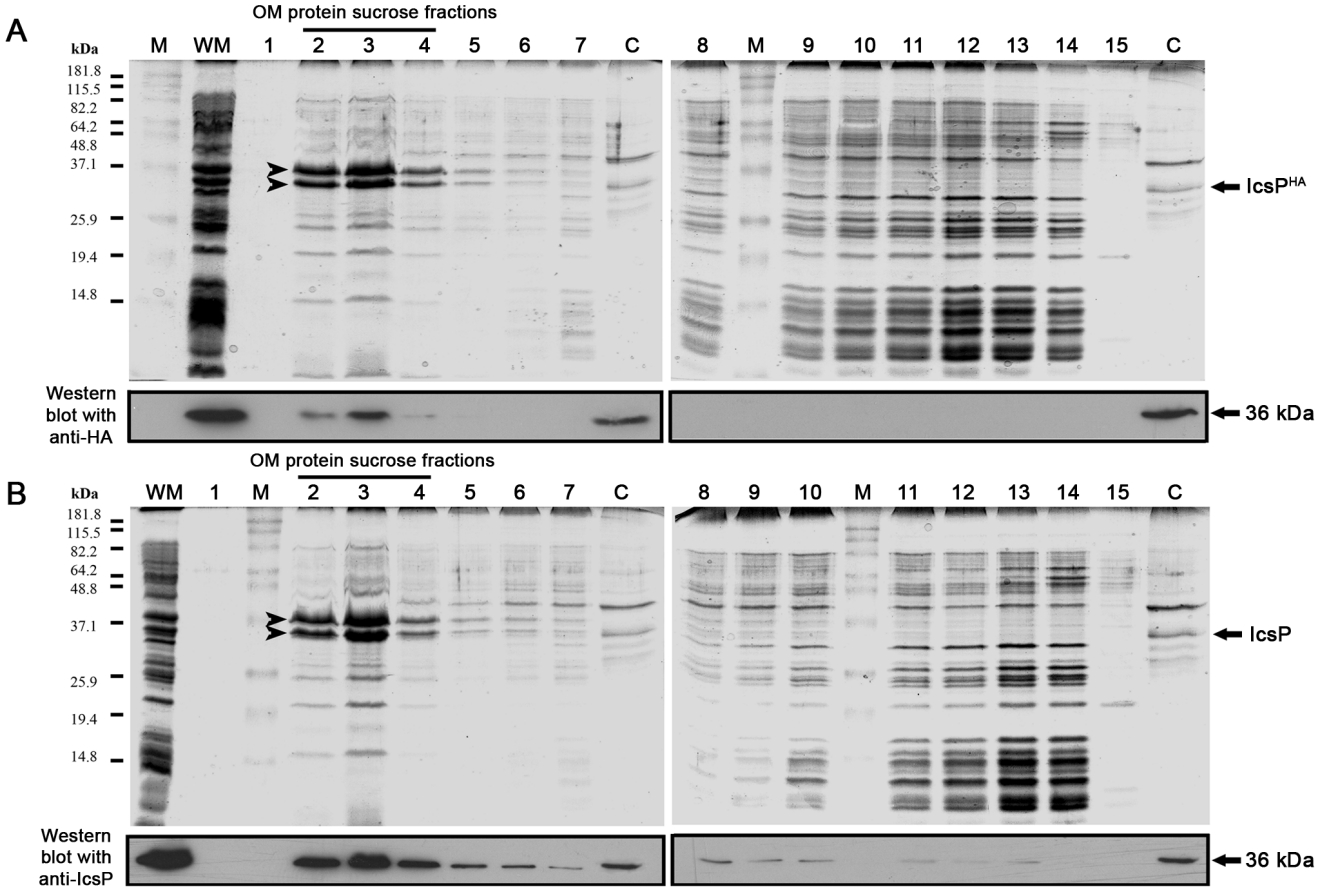
Analysis of IcsP/IcsP^HA^ subcellular localisation by sucrose density gradient centrifugation of WM. *S. flexneri icsP^-^* strains harbouring either pIcsP^HA^ and pIcsP were grown in LB for 1.5 h to an OD_600_ reading of ∼0.4, washed 3 times, and induced with arabinose for 1 h (OD_600_ ∼1). Whole membranes (WM) were prepared by French press lysis, and subjected to sucrose density gradient centrifugation 48] as described in the *Methods*. Fractions (0.5 ml; numbered 1-15) were collected and samples of each were electrophoresed on SDS 15% polyacrylamide gels for staining with Coomassie Blue and Western immunoblotting with anti-HA or anti-IcsP antibodies; (**A**) The results obtained for *icsP^-^* [pIcsP^HA^]; (**B**) The results obtained for *icsP^-^* [pIcsP]. The migration positions of the Benchmark Prestained Marker (M) Standards (Invitrogen) are indicated on the left in kDa. The major OM proteins OmpF+OmpC and OmpA are indicated by the two arrowheads in lane 2. Lane C corresponds to the control lanes containing WC samples of *icsP^-^* [pIcsP^HA^] induced with 0.2% (w/v) arabinose for protein overexpression (positive with both anti-HA and anti-IcsP by Western immunoblotting) as indicated by the arrows on the right. The sucrose fractions in lanes 2 – 4 contain most of the OM proteins, and the sucrose fractions in lanes 8 – 14 contain most of the inner (cytoplasmic) membrane proteins.

IcsP is 60% identical in primary amino acid sequence to the *E. coli* protease OmpT and computer structure modelling predicts that both proteins possess similar β-barrel structures (Fig. 2A). Based on sequence alignments with *E. coli* OmpT (Fig. 2B), a HA epitope tag (YPYDVPDYA) was hence inserted into the OM loop 5 of IcsP (Fig. 2B) using SOE PCR (as described in the *Methods*). An area within this loop region with some sequence diversity between IcsP and OmpT was selected as we reasoned that this sequence variability might allow the protein to accommodate the epitope insertion with little disturbance to the overall structure. The OM loop 5 region was also selected to increase the chance of surface detection by antibodies. OM loops 2 and 4 were not selected for HA tag insertion to avoid the proposed catalytic residues present in OmpT [38] which also exist in IcsP (Fig. 2C). The IcsP^HA^ (as well as IcsP) coding regions were placed in front of the pBAD promoter in pBAD30 [39] to allow expression control with arabinose. Expression of IcsP/IcsP^HA^ was confirmed by Western immunoblotting with anti-IcsP or anti-HA antibodies (Fig. 3A & B, lanes WM and Fig. 1A & C).

### IcsP^HA^ Activity on IcsA

To determine whether insertion of a HA epitope into the OM of IcsP affected IcsP’s protease activity on IcsA, pellet and supernatant protein preparations of 2457T, *icsP^-^* and arabinose-induced *icsP^-^* strains expressing pIcsP^HA^, pIcsP and pBAD30 were subjected to Western immunoblotting with an anti-IcsA antibody. The full length 120 kDa IcsA protein was detected in cell pellet samples of all strains as expected (Fig. 1B, lanes 1, 3, 5, 7 and 9), while the 95 kDa cleaved form of IcsA was only detected in the supernatant sample of 2457T, *icsP^-^* [pIcsP^HA^] and *icsP^-^* [pIcsP] (Fig. 1B, lanes 2, 6 & 8). These results suggest that the insertion of the HA tag into the OM loop 5 of IcsP does not affect its ability to cleave IcsA, and hence the IcsP^HA^ protein is functional.

### Localisation of IcsP/IcsP^HA^ Protein to the OM

Since IcsP^HA^ was modified compared to IcsP and expressed from the pBAD promoter, the presence of IcsP^HA^ protein exclusively in the OM was confirmed by using sucrose density gradient centrifugation. The WM of *icsP^-^* [pIcsP^HA^] and *icsP^-^* [pIcsP] were fractionated into CM and OM on sucrose gradients, and fractions subjected to SDS 15% polyacrylamide gel electrophoresis, prior to visualisation by Coomassie Blue staining and Western immunoblotting with anti-HA and anti-IcsP. Analysis of the sucrose gradient samples showed that fractions which were enriched with OM proteins (OmpF, OmpC, and OmpA) [40] contained the majority of the 36 kDa IcsP^HA^ and IcsP proteins (Fig. 3A & B, lanes 2 to 5). These results indicate that IcsP^HA^ is localised to the OM similar to IcsP, and that the HA insertion did not result in any dramatic disruption of IcsP protein localisation.

### Detection of Arabinose Induced IcsP^HA^ Expression by Western Immunoblotting

Wild-type *S. flexneri* 2457T showed optimal expression levels of native IcsP at 3 h (Fig. 1A). To investigate the conditions required for comparable IcsP^HA^ expression, whole cell lysate samples were prepared from *icsP^-^* [pIcsP^HA^] and *icsP^-^* [pBAD30] induced with 0%, 0.003%, 0.006%, 0.0125%, 0.025%, 0.05%, 0.1% or 0.2% (w/v) arabinose for 1 h. Western immunoblotting with anti-IcsP showed that a band of ∼36 kDa was detected for 2457T and *icsP^-^* [pIcsP^HA^] induced with 0.025%, 0.05%, 0.1% and 0.2% (w/v) (w/v) arabinose (Fig. 1C, lanes 1, 3–6), with expression levels comparable to native IcsP for *icsP^-^* [pIcsP^HA^] observed between 0.025%–0.05% (w/v) arabinose induction (Fig. 1C, lanes 5 & 6). Induction at 0.03% (w/v) arabinose was hence chosen for all subsequent experiments.

### Cell Surface Detection of IcsP^HA^ Distribution in *S. flexneri* 2457T and the Effect of Tunicamycin Treatment

Having established an induction protocol that closely approximates the level of expression of IcsP^HA^ to native IcsP, we next attempted to detect IcsP^HA^ at these levels on the surface of 2457T *icsP^-^* using indirect QD IF microscopy. Strains *icsP^-^* [pIcsP^HA^] and *icsP^-^* [pBAD30] were cultured with 0.03% (w/v) arabinose, fixed, and then probed for HA using a primary anti-HA antibody and a secondary QD 525 conjugated antibody. However, no IcsP^HA^ was detected on the cell surface, and intensity scans of *icsP* ^-^ [pIcsP^HA^] were equivalent to *icsP^-^* [pBAD30] (Fig. S1).

**Figure 4:**
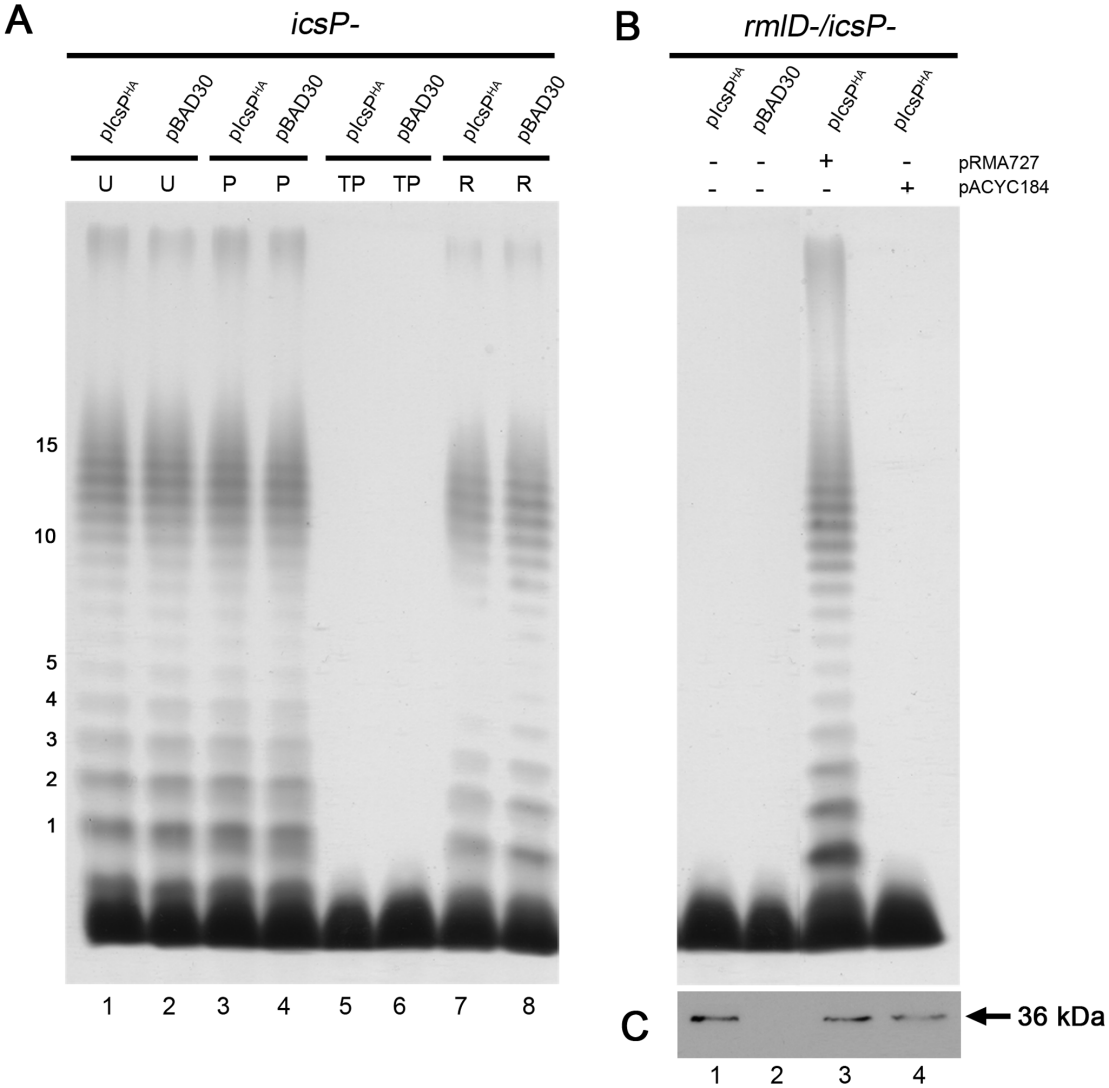
Effect of tunicamycin on the LPS of strains expressing IcsP^HA^. (**A**) Smooth LPS 2457T *icsP^-^* strains harbouring pIcsP^HA^ or pBAD30 were grown to an OD_600_ reading of ∼0.8 in LB, washed 3 times, and treated without TP (U), with PMBN only (P) or with TP treatment for 2 h. Strains were then induced with 0.003% (w/v) arabinose for 1 h, washed 3 times, and grown for an additional 3 h for restoration (R) of LPS Oag; (**B**) Rough LPS 2457T *icsP^-^/rmlD^-^* strains harbouring pIcsP^HA^ and either pRMA727 or pACYC184 (as indicated) were grown to an OD_600_ reading of ∼0.4 in LB, washed 3 times, and induced with arabinose for 1 h. LPS from strains described in (A) and (B) were isolated and detected by silver staining as described in the *Methods*. The first 15 Oag RUs are indicated on the side of each gel. Each lane contains ∼2 x 10^8^ bacterial cells of each strain; (**C**) Western blots on whole cell lysates obtained from strains in (B) were probed with rabbit anti-IcsP antiserum. The size of the full length IcsP^HA^ protein (∼36 kDa) is indicated. Each lane contains 5 x 10^7^ bacterial cells of each strain.

Since IcsP^HA^ could not be detected on the cell surface of *icsP^-^* [pIcsP^HA^], we reasoned that the presence of LPS Oag may mask the detection of surface IcsP as this has previously been shown for IcsA [21]. An LPS Oag depletion-regeneration assay [29] was hence carried out on *icsP^-^* [pIcsP^HA^] and *icsP^-^* [pBAD30] induced with 0.03% (w/v) arabinose, followed by IF labelling. This assay involves the use of an inhibitor of the WecA enzyme necessary for Oag subunit biosynthesis using tunicamycin, and polymyxin B nonapeptide (PMBN) was used to improve OM penetration. Upon removal of these two chemicals from growing bacteria, LPS Oag is regenerated [29]. Analysis of the resulting LPS by SDS-PAGE and silver staining showed that *icsP^-^* [pIcsP^HA^] and *icsP^-^* [pBAD30] samples treated with tunicamycin/PMBN (TP) had depleted LPS Oag (Fig. 4A, lanes 5 & 6), with Oag production restored (R) upon removal of TP (Fig. 4A, lanes 7 & 8). Untreated (U) and PMBN treated (P) samples showed no inhibition of Oag biosynthesis as expected (Fig. 4A, lanes 1–4).

**Figure 5:**
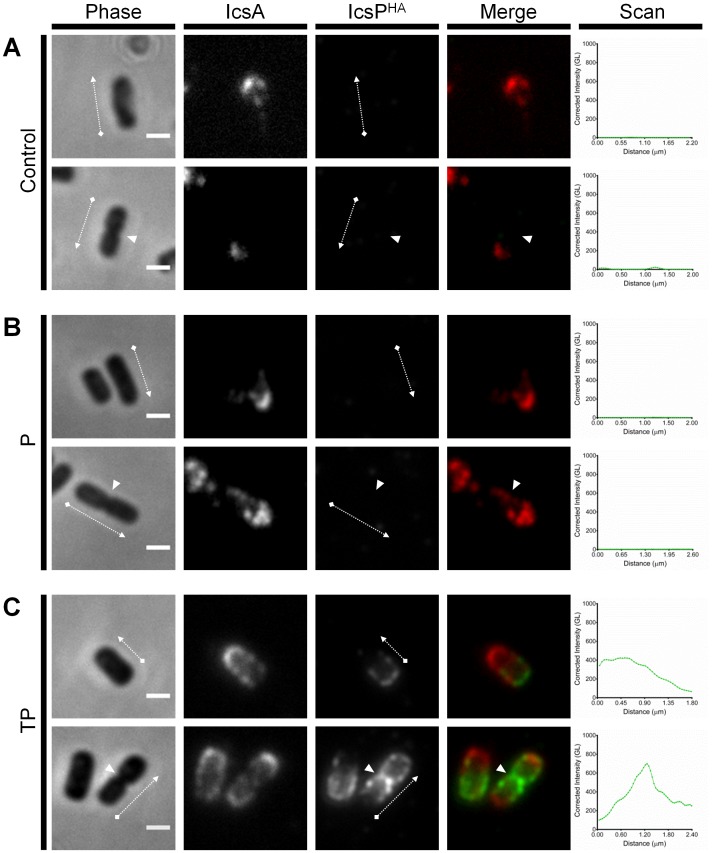
Effect of LPS Oag-depletion in detection of surface IcsP^HA^. Smooth LPS 2457T *icsP^-^* strains harbouring pIcsP^HA^ were subcultured in LB broth to an OD_600_ reading of ∼0.8, washed 3 times in LB, and then further cultured for 2 h; (**A**) in the absence of TP, (**B**) in the presence of PMBN only, or (**C**) in the presence of TP. Arabinose was included in the final hour of treatment at a concentration of 0.03% (w/v). Samples were then fixed and subjected to QD IF using antibodies to HA epitope and IcsA. Non-septating and septating cells (upper and lower rows respectively) are shown for each treatment group. Representative bacteria are shown. Scan  =  Single line-scans measuring the fluorescence intensity of IcsP^HA^ detected along the surface of the bacterium, Bars  =  1 µm, Arrows  =  direction of line-scan, Arrow heads  =  septa, Control  =  grown in absence of both tunicamycin and PMBN, P  =  PMBN, TP  =  tunicamycin/PMBN, GL  =  Grey level, Phase  =  phase contrast image, IcsP^HA^  =  image of fluorescence at 525 nm, IcsA  =  image of fluorescence at 625 nm, Merge  =  overlay of IcsP^HA^ and IcsA images.

Following successful depletion of LPS Oag, QD IF microscopy was performed on the above mentioned samples. IcsP^HA^ could not be detected on untreated or PMBN treated *icsP* ^-^ [pIcsP^HA^] cells, as expected (Fig. S2A). However, surface IcsP^HA^ was detected on *icsP* ^-^ [pIcsP^HA^] cells treated with TP, suggesting that LPS Oag is able to mask antibody accessibility to IcsP^HA^ (Fig. S2A). Interestingly, IcsP^HA^ appeared to be distributed asymmetrically over the cell surface of the majority of cells examined and fluorescence intensity line-scans revealed that IcsP^HA^ localised preferentially to one pole of non-septating cells and the septa of septating cells (Fig. S2A). To determine if the distribution of IcsP^HA^ on the cell surface segregated to either the new pole or the old pole, additional staining of the bacteria was conducted with anti-IcsA antibodies since IcsA is known to localise to the old cell pole [5]. Again, IcsP^HA^ could not be detected on untreated or PMBN treated *icsP* ^-^ [pIcsP^HA^] cells (Fig. 5A & B) but could be detected after TP treatment (Fig. 5C). Peak IcsP^HA^ detection was consistently observed at the new pole (opposing IcsA at the old pole) and at the septum of septating bacteria as shown by line-scans (Fig. 5C). As expected, no surface IcsP^HA^ was detected for untreated, PMBN treated, or TP treated samples of *icsP^-^* [pBAD30] when stained for IcsP^HA^ (Fig. S2B) or double stained for IcsP^HA^ and IcsA (Fig. S3). Notably, IcsA on *icsP^-^* [pBAD30] was detected at higher amounts laterally and at the septa of bacteria (Fig. S3) as previously seen for *ΔicsP* strains [13].

### Cell Surface Detection of IcsP^HA^ Distribution in Rough LPS *S. flexneri* 2457T

To further investigate the effect of Oag on IcsP distribution, a 2457T *icsP^−^/rmlD^-^* double mutant was constructed to independently assess the distribution of IcsP^HA^ in a rough LPS background. Mutation of the *rmlD* gene in *S. flexneri* results in a strain which is unable to synthesise the precursor deoxythymidine diphosphate (dTDP)- rhamnose required for Oag repeat units and, hence, results in a rough LPS phenotype [29], [41]. Analysis of the resulting LPS conferred by *icsP^−^/rmlD^-^* strains expressing pIcsP^HA^ and pBAD30 showed that rough LPS was observed for both (Fig. 4B, lanes 1 & 2), with a band consistent with the size of the IcsP^HA^ protein (36 kDa) detected only in the *icsP^−^/rmlD^-^* [pIcsP^HA^] sample by Western immunoblotting, as expected (Fig. 4C, lane 1).

**Figure 6:**
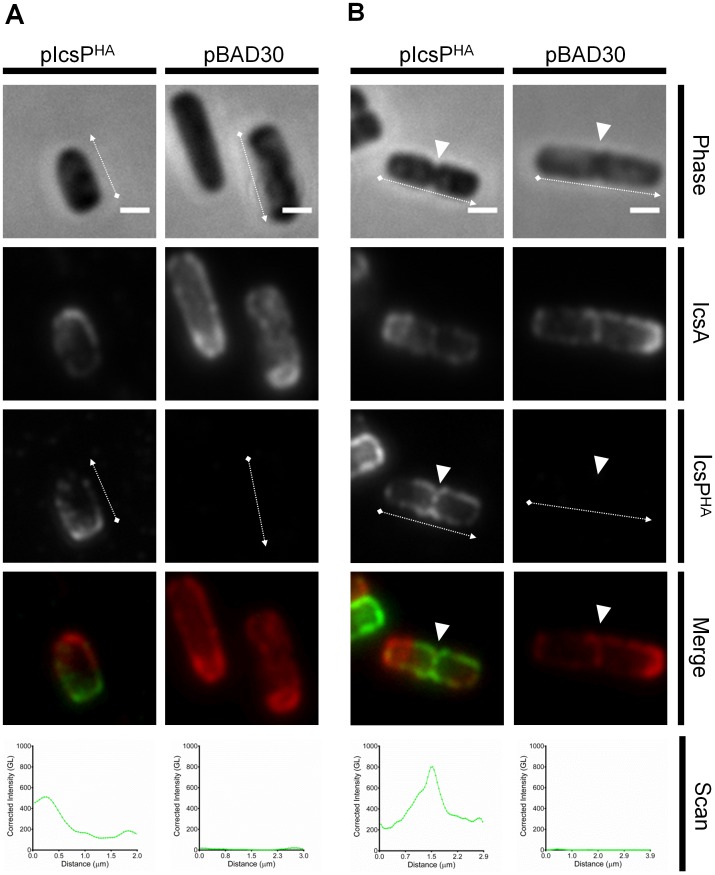
Localisation of IcsP^HA^ on the surface of rough LPS 2457T *icsP^-^/rmlD^-^*. Rough LPS 2457T *icsP^-^/rmlD^-^* strains harbouring pIcsP^HA^ (left columns) or pBAD30 (right columns) were subcultured in LB broth for 1.5 h to an OD_600_ reading of ∼0.4. Cultures were then washed 3 times in LB, induced with 0.03% (w/v) arabinose for 1 h, fixed, and subjected to QD IF using antibodies for HA epitope and IcsA. Non-septating and septating life stages are shown in **A** and **B** respectively. Representative bacteria are shown. Scan  =  Single line-scans measuring the fluorescence intensity of IcsP^HA^ detected along the surface of the bacterium, Bars  =  1 µm, Arrows  =  direction of line-scan, Arrow heads  =  septa, GL  =  Grey level, Phase  =  phase contrast image, IcsP^HA^  =  image of fluorescence at 525 nm, IcsA  =  image of fluorescence at 625 nm, Merge  =  overlay of IcsP^HA^ and IcsA images.

Fixed samples of *icsP^−^/rmlD^-^* [pIcsP^HA^] and *icsP^−^/rmlD^-^* [pBAD30] cells were then probed for both IcsP^HA^ and IcsA using the same QD IF staining protocol as previously conducted for smooth strains. Similarly to LPS Oag-depleted *icsP* ^-^ [pIcsP^HA^], IcsP^HA^ was detected on the bacterial surface most predominately at the new pole (Fig. 6A) and the septum (Fig. 6B). Again, the majority of septating cells had higher peak IcsP^HA^ intensity at the septum than new poles of non-septating cells (line-scans Fig. 6A & B). Single staining of these cells for IcsP^HA^ was also conducted and yielded the same localisation results (Fig. S4). As expected, IcsP^HA^ was not detected on the *icsP^−^/rmlD^-^* [pBAD30] strain in IF microscopy experiments when either single (Fig. S4) or double stained (Fig. 6B). Again, IcsA on *icsP^−^/rmlD^-^* [pBAD30] was detected at higher amounts laterally and at the septum (Fig. 6A & B).

**Figure 7:**
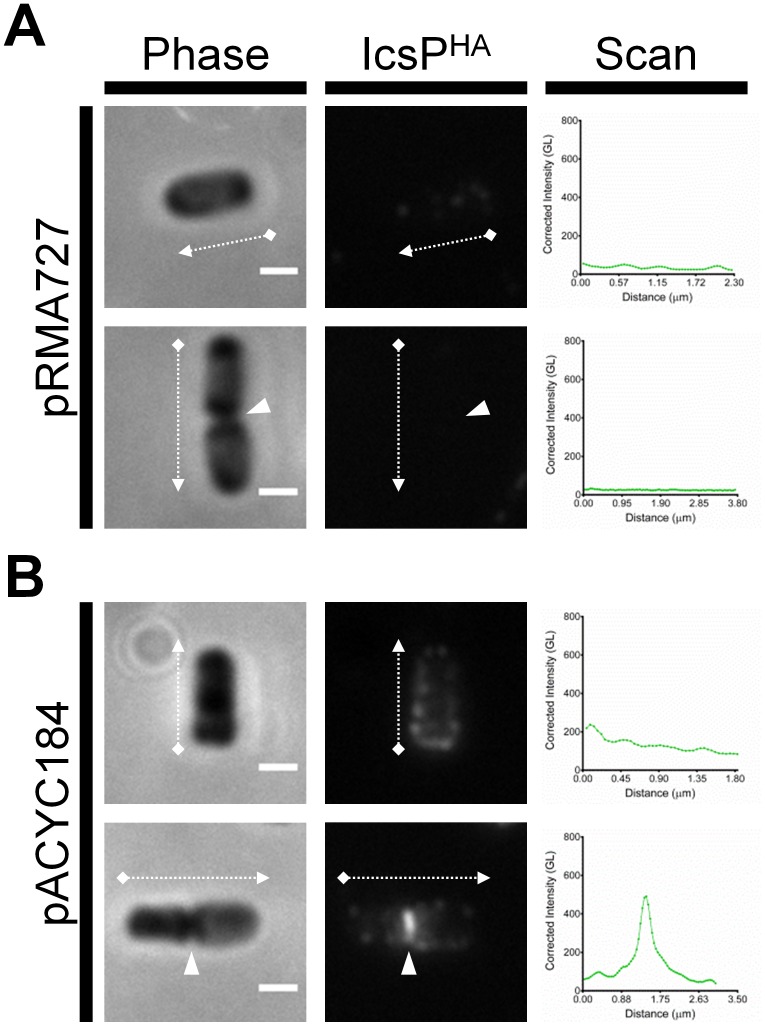
Complementation of *rmlD* restores LPS masking effect on IcsP^HA^ detection. Rough LPS 2457T *icsP^-^/rmlD^-^* strains harbouring pIcsP^HA^, and either (**A**) pRMA727 or (**B**) pACYC184, were subcultured in LB broth for 1.5 h to an OD_600_ reading of ∼0.4. Cultures were then washed 3 times in LB, induced with 0.03% (w/v) arabinose for 1 h, fixed, and subjected to QD IF using antibodies for the HA epitope. Representative bacteria are shown. Scan  =  Single line-scans measuring the fluorescence intensity of IcsP^HA^ detected along the surface of the bacterium, Bars  =  1 µm, Arrows  =  direction of line-scan, Arrow heads  =  septa, GL  =  Grey level, Phase  =  phase contrast image, IcsP^HA^  =  image of fluorescence at 525 nm.

To again demonstrate the effect of LPS Oag masking of IcsP^HA^, smooth LPS structure was restored in the *icsP^−^/rmlD^-^* strain expressing pIcsP^HA^ by transforming pRMA727 carrying a functional *rmlD* gene. Analysis of the resulting LPS conferred by *icsP^−^/rmlD^-^*[pIcsP^HA^][pRMA727] showed restored smooth LPS phenotype (Fig. 4B, lane 3), and when probed for IcsP^HA^ by QD IF microscopy, was barely detectable (Fig. 7A). The control *icsP^−^/rmlD^-^* [pIcsP^HA^] strain carrying pACYC184 conferred a rough LPS phenotype (Fig. 4B, lane 4) and IcsP^HA^ was detected by IF microscopy with the same distribution observed previously (Fig. 7B).

### Multi-cell Line-scan Analysis of IcsP^HA^ Surface Distribution

**Figure 8:**
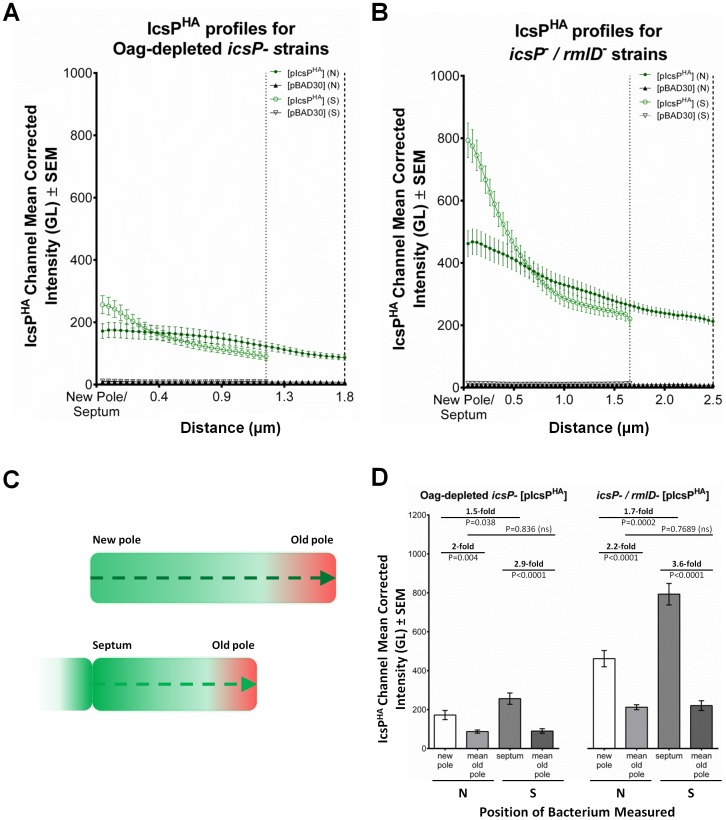
Quantification and statistical analysis of IcsP^HA^ surface distribution. A total of 9 independent cultures of Smooth LPS 2457T *icsP^-^* strains harbouring pIcsP^HA^ or pBAD30 were subcultured in LB for 1.5 h to an OD_600_ reading of ∼0.4, washed 3 times in LB, and then further cultured for 2 in the presence of TP for Oag-depletion. Arabinose was included in the final hour of treatment at a concentration of 0.03% (w/v). A total of 9 independent cultures of Rough LPS 2457T *icsP^-^/rmlD^-^* strains harbouring pIcsP^HA^ or pBAD30 were also subcultured LB broth to an OD_600_ reading of ∼0.4, washed 3 times in LB, and induced with 0.03% (w/v) arabinose for 1 h. A sample from each culture was then fixed and subjected to QD IF using antibodies for HA epitope and IcsA. Fluorescence intensity scans of IcsP^HA^ (525 nm wavelength) were conducted on multiple bacteria from each independent sample and accumulated scans were used to create mean intensity profiles. A total 450 non-septating bacteria were scanned for each strain. A further 355, 256, 172, and 173 septating bacteria were scanned for Oag-depleted *icsP^-^* [pIcsP^HA^], Oag-depleted *icsP^-^* [pBAD30], *icsP^-^/rmlD^-^* [pIcsP^HA^], and *icsP^-^/rmlD^-^* [pBAD30] respectively. Resultant IcsP^HA^ mean surface profiles for (**A**) Oag-depleted *icsP^-^* and (**B**) *icsP^-^/rmlD^-^* bacteria are shown. Dotted and dashed vertical lines indicate mean positions of old poles for septating and non-septating bacteria respectively. The schematic (**C**) shows methodology of IcsP^HA^ intensity scan directions from either new poles to old poles (as marked by IcsA) of non-septating bacteria, or septa to old poles of septating bacteria. Student’s two-tailed t-tests (**D**) were also conducted on the fold differences of mean IcsP^HA^ intensities between discrete positions (new pole, old pole, and septum) of Oag-depleted *icsP^-^* and *icsP^-^/rmlD^-^* bacteria. SEM  =  standard error of the mean, GL  =  Grey level, N  =  non-septating cells, S  =  septating cells, P  =  p-value, ns  =  not significant.

To determine if the observed sub-cellular preference of IcsP^HA^ to the new poles and septa is a statistically significant phenomenon, cumulative QD IF line-scan analyses on both IcsP^HA^/IcsA double stained LPS Oag-depleted *icsP* ^-^ strains and rough *icsP^−^/rmlD^-^* strains was conducted for a larger population of cells. Scanning was initiated from the new pole to the old pole (as marked by IcsA) for non-septating cells, and from the septum to the old pole for septating cells (Fig 8C). From 9 independent samples of LPS Oag-depleted *icsP^-^* [pIcsP^HA^] and *icsP^-^* [pBAD30], a total of 450 non-septating cells each were line-scanned. Additionally a total of 355 and 256 septating cells were scanned for *icsP^-^* [pIcsP^HA^] and *icsP^-^* [pBAD30] respectively. The resultant mean fluorescence intensity profiles show that IcsP^HA^ is preferentially localised at the new pole and tends to gradually decrease towards the old pole on non-septating cells expressing pIcsP^HA^ (Fig. 8A). For septating cells, IcsP^HA^ mean intensity is localised highest at the septum and declines more steeply towards the old pole (Fig. 8A). As expected, the intensity profiles of *icsP^-^* [pBAD30] cells were at a negligible level (Fig. 8A). Statistical analysis of mean IcsP^HA^ intensity at discrete bacterial positions of LPS-depleted *icsP^-^* [pIcsP^HA^] confirmed that the localisation of IcsP^HA^ is: (i) 2-fold higher at the new pole of non-septating cells than the old pole (P = 0.004), (ii) 2.9-fold higher at the septum of septating cells than the old pole (P<0.0001), and (iii) 1.5-fold higher at septa of septating cells compared to new poles of non-septating cells (P = 0.038) (Fig. 8D).

For the rough LPS strains, 9 independent samples of *icsP^−^/rmlD^-^* [pIcsP^HA^] and *icsP^−^/rmlD^-^* [pBAD30] were investigated with a total of 450 non-septating cells each line-scanned. Additionally, a total of 172 and 173 septating cells were scanned for *icsP^−^/rmlD^-^* [pIcsP^HA^] and *icsP^−^/rmlD^-^* [pBAD30] respectively. Again, the mean intensity profiles of non-septating cells expressing pIcsP^HA^ shows IcsP^HA^ is preferentially localised at the new pole and tends to gradually decrease towards the old pole (Fig. 8B). Likewise, IcsP^HA^ mean intensity of septating cells is localised highest at the septum and declines very steeply towards the old pole (Fig. 8B). As expected, the mean intensity profiles of *icsP^−^/rmlD^-^* [pBAD30] were at a negligible level (Fig. 8B). Statistical analysis of mean IcsP^HA^ intensity at discrete bacterial positions of *icsP^−^/rmlD^-^* [pIcsP^HA^] again confirmed that the localisation of IcsP^HA^ is: (i) 2.2-fold higher at the new pole of non-septating cells than the old pole (P<0.0001), (ii) 3.6-fold higher at the septum of septating cells than the old pole (P<0.0001), and (iii) 1.7-fold higher at septa of septating cells compared to new poles of non-septating cells (P = 0.0002) (Fig. 8D). Although, IcsP^HA^ fluorescence intensity was an average of 2.5 times higher on *icsP^−^/rmlD^-^* [pIcsP^HA^] cells than LPS-depleted *icsP^-^* [pIcsP^HA^] cells, the ratios of intensity between bacterial positions were comparable to the respective fold-changes observed for LPS Oag-depleted *icsP^-^* [pIcsP^HA^], suggesting that IcsP^HA^ is localised similarly in both types of Oag deficient cells. The lower level of IcsP^HA^ detection on LPS-depleted *icsP^-^* [pIcsP^HA^] compared to *icsP^−^/rmlD^-^* [pIcsP^HA^] indicates that TP treatment is not 100% efficient in inhibiting Oag synthesis.

## Discussion

The cell surface distribution of neither IcsP, or any other member of the Omptin family, had not been previously determined. In this study we investigated the distribution of IcsP on the cell surface of *S. flexneri* 2a 2457T using a HA-tagged IcsP protein (Fig. 2) under pBAD control. Characterisation of IcsP^HA^ showed that it was functionally able to cleave IcsA, and was secreted into the OM comparably to IcsP (Fig. 1B & 3). However, when IcsP^HA^ was expressed at native IcsP equivalent levels (Fig. 1A & C), it was undetectable in the OM via QD IF microscopy in smooth LPS *S. flexneri* (Fig. S1) but detectable on both LPS Oag-depleted and rough LPS *Shigella* bacteria (Fig. 4, 5 & 6). Furthermore, this masking effect was restored in rough strains upon complementation of *rmlD* (Fig. 4B & 7). We suggest that the long LPS Oag chains of smooth strains sterically hinder the accessibility of antibodies to the OM surface. This type of protein masking by LPS Oag has also been shown for IcsA in 2457T [21], and several other OM proteins [22], [23]. It is interesting to note that multi-cell line-scan analysis of IcsP^HA^ detection on the cell surface showed that IcsP^HA^ fluorescence intensity was higher for rough LPS cells than for LPS Oag-depleted cells (Fig. 8). Similar to its Omptin homolog, OmpT, IcsP possesses most of the putative LPS-binding sites found in OmpT (Fig. 2C) [38] and may also interact with LPS. Since TP is only partly effective at blocking LPS Oag synthesis, it is possible that the few LPS Oag molecules synthesised are still closely bound to IcsP. This may cause a small amount of masking and may explain the lower fluorescence intensity observed on LPS Oag-depleted cells (Fig. 8A) compared to the rough LPS cells (Fig. 8B).

**Figure 9:**
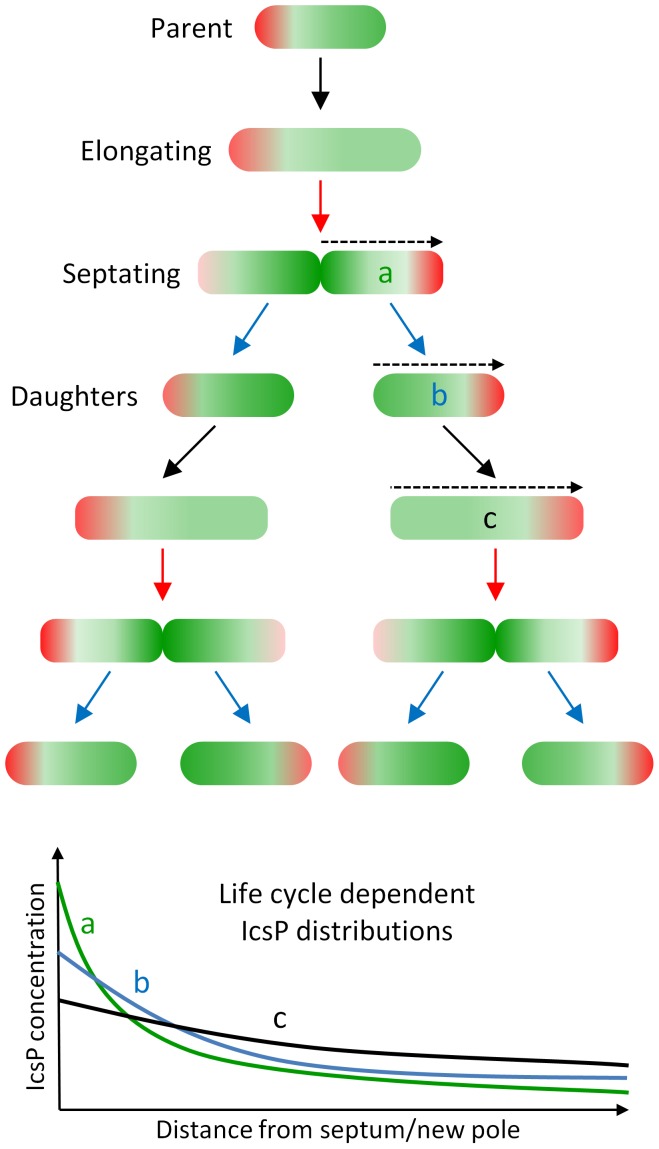
Model for differential inheritance of spatially separated OM proteins IcsA and IcsP. Successive generations of *S. flexneri* are shown including: septating cells, newly formed daughters, and elongating cells. Solid black, red, and blue arrows denote the events of growth/elongation, septation, and division respectively. IcsA localisation is represented by red shading and IcsP by green shading where the darker the shading indicates higher protein concentration in the OM. The bottom graph represents IcsP distributions and gradients that result from hypothetical line-scans of: (a) a septating cell, (b) a newly formed daughter cell, and (c) an elongated cell from this model. IcsP on septating cells (a) declines steeply from the septum towards the old pole, whereas it’s gradient on elongated cells (b) is more moderate between poles. A newly formed cell (c) would have an intermediate gradient. Dotted arrow  =  direction of hypothetical line-scan.

IcsA distributions in *ΔicsP* strains previously provided indirect evidence to suggest that IcsP is distributed equally over the cell surface of *S. flexneri* [12], [13], [42]. However, once the masking effect of LPS Oag was circumvented, we were able to extensively study the distribution profile of IcsP^HA^ in the OM. This work has shown that IcsP^HA^ (and likely IcsP) has an asymmetrical distribution which may be cell cycle dependent. On dividing cells IcsP accumulates at high concentration at the septum and then declines steeply towards the old pole of the emerging daughter cell. However, once divided, detection of IcsP at the new pole (the pole derived from the septum) of daughter cells decreased significantly and then the decline towards the old pole is more moderate. To explain this, we propose that IcsP is directed to septa of dividing cells and that daughter cells ‘inherit’ a higher concentration of IcsP at new poles (model Fig. 9). As the daughter cell elongates, IcsP is laterally diluted, resulting in the gentle gradient on non-septating cells. In this model, lateral dilution of IcsP may occur by membrane insertion events during cell elongation similarly to that described for *E. coli* LamB [43]. This model predicts increased cleavage of IcsA at the septum and lateral regions of the cell in order to help set up a relatively higher concentration of IcsA at the old pole and to maintain the old pole preference of IcsA in daughter cells. Additionally, the model may explain, and also fits, the common observation that IcsA distributions and intensities vary between cells in a given population of the same strain.

Many proteins are known to accumulate at the inner membrane of the mid-cell in order to mediate septum formation and cell division – for instance, FtsZ polymerises at the cytoplasmic face to form the Z-ring and attract further divisome components [44]. However, few OM proteins have been shown to concentrate to the septum. A notable exception to this is the OM lipoprotein LpoB which localises as distinct foci at septa and complexes with periplasmic penicillin binding protein 1B tethering it to other divisome components [44], [45]. Interestingly, septal localisation of LpoB is lost when peptidoglycan synthesis is inhibited [45]. Whether the localisation of integral OM protein IcsP to the septal OM requires similar interactions with divisome components, or is dependent on peptidoglycan synthesis, remains to be investigated.

In summary, this work has shown for the first time the surface localisation of IcsP, a member on the Omptin family of OM proteases. We have observed that: (i) the distribution of IcsP is masked by LPS Oag in *S. flexneri* 2457T, and (ii) IcsP is concentrated at new poles and at the septum of dividing cells. This distribution of IcsP explains the observed IcsA localisation defect in *ΔicsP* strains [11], [12]. We have also proposed a model to explain the inheritance of OM proteins IcsP and IcsA through generations of cell division. Finally, unmasking of surface antigens via LPS Oag-depletion may be useful in the study of other minimally exposed OM proteins.

## Supporting Information

Figure S1 **Inability to detect IcsP^HA^ on the cell surface of 2457T** ***icsP^-^*** **.** Smooth LPS 2457T *icsP^-^* strains expressing pIcsP^HA^ (left column) or pBAD30 (right column) were subcultured in LB for 1.5 h to an OD_600_ reading of ∼0.4. Cultures were then washed 3 times in LB, induced with 0.03% (w/v) arabinose for 1 h, fixed, and subjected to QD IF using antibodies for HA epitope and IcsA. Representative bacteria are shown. Scan = Single line-scans measuring the intensity of IcsP^HA^ detected along the surface of the bacterium, Bars = 1 µm, Arrows = direction of line-scan, GL = Grey level, Phase = phase contrast image, IcsP^HA^ = image of fluorescence at 525 nm.
(TIF)

Figure S2 **Single IcsP^HA^ staining of LPS-depleted 2457T** ***icsP^-^*** **.** Smooth LPS 2457T *icsP^-^* strains harbouring (**A**) pIcsP^HA^ or (**B**) pBAD30 were subcultured in LB broth for 1.5 h to an OD_600_ reading of ∼0.4, washed 3 times in LB, and then further cultured for 2 h in either: the absence of TP, in the presence of PMBN only, or in the presence of TP. Arabinose was included in the final hour of treatment at a concentration of 0.03% (w/v). Samples were then fixed and subjected to QD IF using antibodies to HA epitope. Representative non-septating and septating cells are shown for each treatment group. Scan = Single line-scans measuring the fluorescence intensity of IcsP^HA^ detected along the surface of the bacterium, Bars = 1 µm, Arrows = direction of line-scan, Arrow heads = septa, Control = grown in absence of both tunicamycin and PMBN, P = PMBN, TP = tunicamycin/PMBN, GL = Grey level, Phase = phase contrast image, IcsP^HA^ = image of fluorescence at 525 nm
(TIF)

Figure S3 **Double stained IF of LPS depleted 2457T** ***icsP^-^*** **[pBAD30].** Smooth LPS 2457T *icsP^-^* strains harbouring pBAD30 were subcultured in LB broth for 1.5 h to an OD_600_ reading of ∼0.4, washed 3 times in LB, and then further cultured for 2 h; (**A**) in the absence of TP, (**B**) in the presence of PMBN only, or (**C**) in the presence of TP. Arabinose was included in the final hour of treatment at a concentration of 0.03% (w/v). Samples were then fixed and subjected to QD IF using antibodies to HA epitope and IcsA. Non-septating and septating cells (upper and lower rows respectively) are shown for each treatment group. Representative bacteria are shown. Scan = Single line-scans measuring the fluorescence intensity of IcsP^HA^ detected along the surface of the bacterium, Bars = 1 µm, Arrows = direction of line-scan, Arrow heads = septa, Control = grown in absence of both tunicamycin and PMBN, P = PMBN, TP = tunicamycin/PMBN, GL = Grey level, Phase = phase contrast image, IcsP^HA^ = image of fluorescence at 525 nm, IcsA = image of fluorescence at 625 nm, Merge = overlay of IcsP^HA^ and IcsA images.
(TIF)

Figure S4 **Single IcsP^HA^ staining of 2457T** ***icsP^−^/rmlD^-^*** **.** Rough LPS 2457T *icsP^−^/rmlD^-^* strains harbouring pIcsP^HA^ (left columns) or pBAD30 (right columns) were subcultured LB broth for 1.5 h to an OD_600_ reading of ∼0.4. Cultures were then washed 3 times in LB, induced with 0.03% (w/v) arabinose for 1 h, fixed, and subjected to QD IF using antibodies for HA epitope. Non-septating and septating life stages are shown in **A** and **B** respectively. Representative bacteria are shown. Scan = Single line-scans measuring the fluorescence intensity of IcsP^HA^ detected along the surface of the bacterium, Bars = 1 µm, Arrows = direction of line-scan, Arrow heads = septa, GL = Grey level, Phase = phase contrast image, IcsP^HA^ = image of fluorescence at 525 nm.
(TIF)
