# Correction: LPS Unmasking of *Shigella flexneri* Reveals Preferential Localisation of Tagged Outer Membrane Protease IcsP to Septa and New Poles

**DOI:** 10.1371/journal.pone.0111656

**Published:** 2014-10-16

**Authors:** The PLOS ONE Staff

There are errors in Figure 1, “Detection of IcsP/IcsPHA expression and activity on IcsA by Western immunoblotting.” Please see the corrected Figure 1 here.

**Figure 1:**
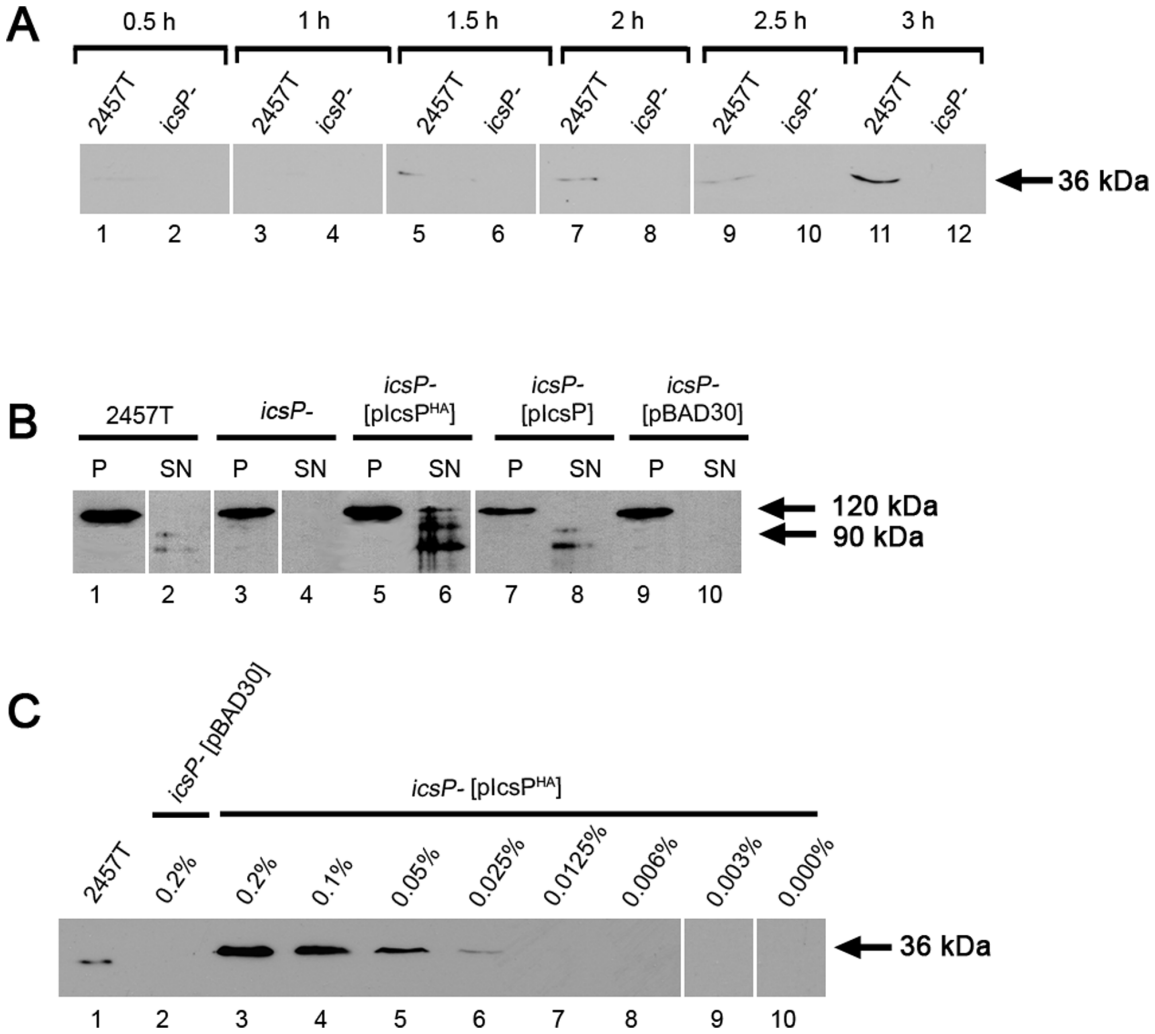
Detection of IcsP/IcsP^HA^ expression and activity on IcsA by Western immunoblotting. (A) *S. flexneri* strains 2457T and 2457T *icsP^-^* mutant (*icsP^-^*) were grown in LB and whole cell lysate samples were taken at 0.5, 1, 1.5, 2, 2.5 and 3 h after subculture, followed by electrophoresis on a SDS 15 % polyacrylamide gel and Western immunoblotting with rabbit anti-IcsP antiserum; (B) *S. flexneri* strains 2457T, *icsP^-^*and *icsP^-^* harbouring pIcsP, pIcsP^HA^ or pBAD30 (as indicated) were grown in LB for 1.5 h to an OD600 reading of ∼0.4, washed 3 times, and induced with arabinose for 1 h. Pellet and supernatant protein samples were then prepared and electrophoresed on a SDS 15 % polyacrylamide gel, followed by Western immunoblotting with rabbit anti-IcsA antibodies. The size of the full length IcsA protein (120 kDa) and the cleaved form of IcsA (95 kDa) are indicated; (C) *S. flexneri* strains 2457T and *icsP^-^* harbouring pIcsP^HA^ or pBAD30 were grown in LB as described in (B), followed by induction with 0%, 0.003%, 0.006%, 0.0125%, 0.025%, 0.05%, 0.1% or 0.2% (w/v) arabinose for 1 h. Whole cell lysates were prepared and electrophoresed on a SDS 15 % polyacrylamide gel, followed by Western immunoblotting with rabbit anti-IcsP antiserum. The size of the full length IcsP protein (36 kDa) is indicated in (A) and (C). Each lane contains 5 x 10^7^bacterial cells of each strain.

There are errors in Figure 4, “Effect of tunicamycin on the LPS of strains expressing IcsP^HA^.” Please see the corrected Figure 4 here.

**Figure 4:**
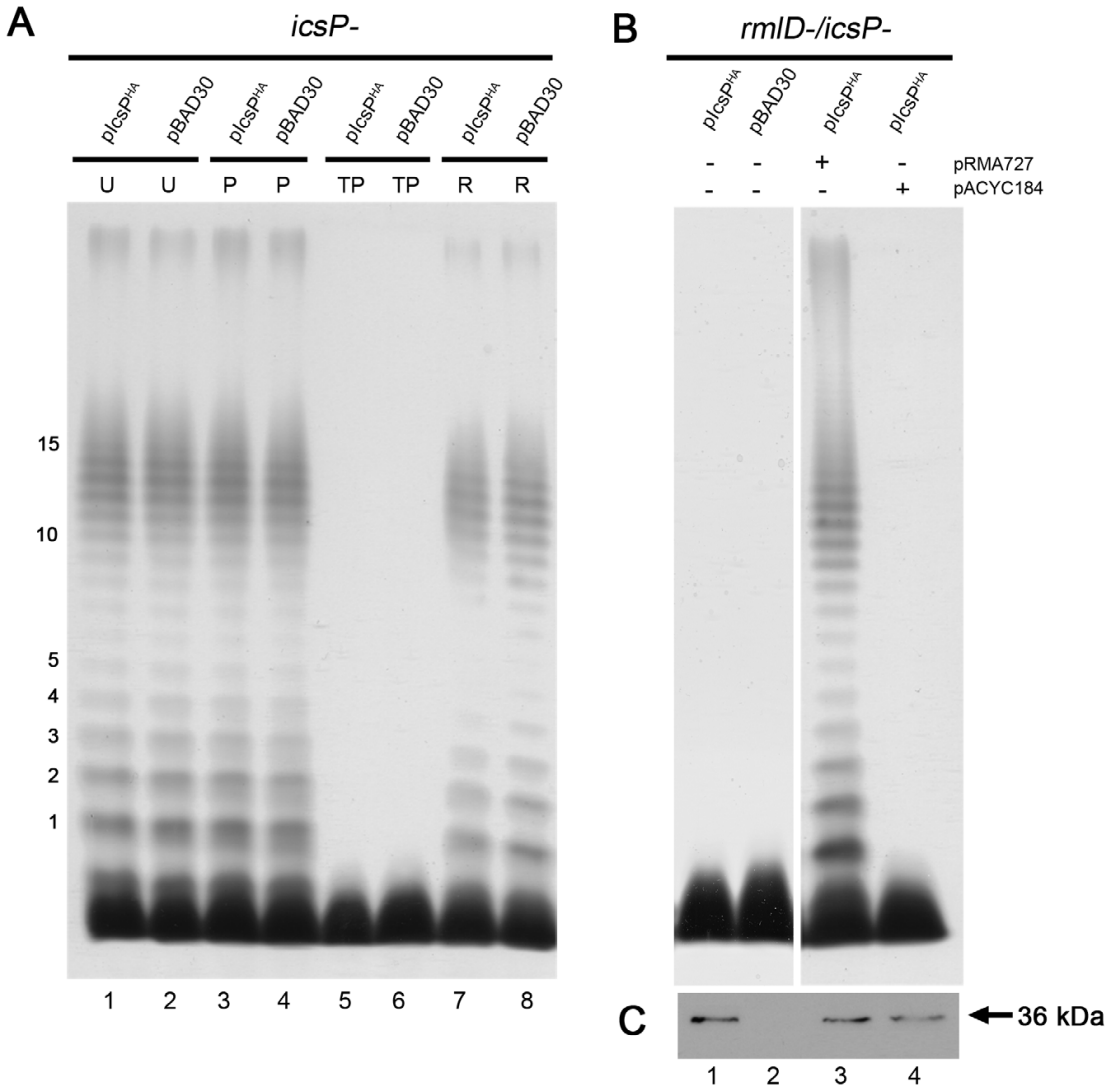
Effect of tunicamycin on the LPS of strains expressing IcsP^HA^. (A) Smooth LPS 2457T *icsP^-^* strains harbouring pIcsP^HA^ or pBAD30 were grown to an OD_600_ reading of ∼0.8 in LB, washed 3 times, and treated without TP (U), with PMBN only (P) or with TP treatment for 2 h. Strains were then induced with 0.003% (w/v) arabinose for 1 h, washed 3 times, and grown for an additional 3 h for restoration (R) of LPS Oag; (B) Rough LPS 2457T *icsP^-^/rmlD^-^* strains harbouring pIcsP^HA^ and either pRMA727 or pACYC184 (as indicated) were grown to an OD_600_ reading of ∼0.4 in LB, washed 3 times, and induced with arabinose for 1 h. LPS from strains described in (A) and (B) were isolated and detected by silver staining as described in the *Methods*. The first 15 Oag RUs are indicated on the side of each gel. Each lane contains ∼2 x 10^8^ bacterial cells of each strain; (C) Western blots on whole cell lysates obtained from strains in (B) were probed with rabbit anti-IcsP antiserum. The size of the full length IcsP^HA^ protein (∼36 kDa) is indicated. Each lane contains 5 x 10^7^ bacterial cells of each strain.

There are errors in Figure 5, “Effect of LPS Oag-depletion in detection of surface IcsP^HA^.” Please see the corrected Figure 5 here.

**Figure 5:**
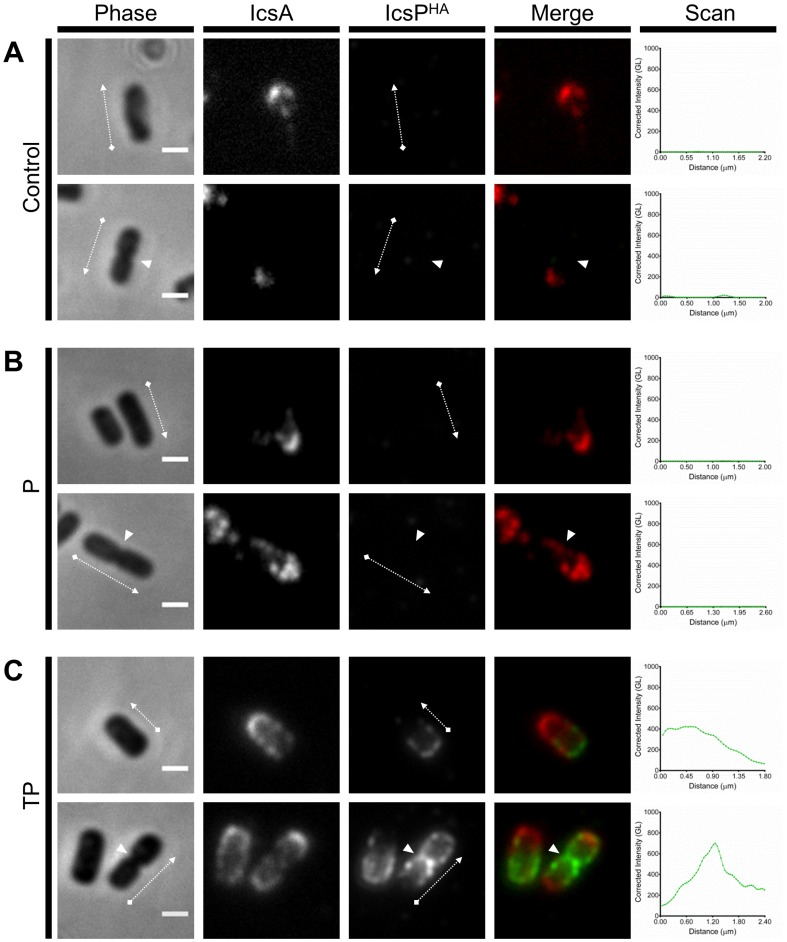
Effect of LPS Oag-depletion in detection of surface IcsP^HA^. Smooth LPS 2457T *icsP^-^* strains harbouring pIcsP^HA^ were subcultured in LB broth to an OD_600_ reading of ∼0.8, washed 3 times in LB, and then further cultured for 2 h; (A) in the absence of TP, (B) in the presence of PMBN only, or (C) in the presence of TP. Arabinose was included in the final hour of treatment at a concentration of 0.03% (w/v). Samples were then fixed and subjected to QD IF using antibodies to HA epitope and IcsA. Non-septating and septating cells (upper and lower rows respectively) are shown for each treatment group. Representative bacteria are shown. Scan  =  Single line-scans measuring the fluorescence intensity of IcsP^HA^ detected along the surface of the bacterium, Bars  =  1 µm, Arrows  =  direction of line-scan, Arrow heads  =  septa, Control  =  grown in absence of both tunicamycin and PMBN, P  =  PMBN, TP  =  tunicamycin/PMBN, GL  =  Grey level, Phase  =  phase contrast image, IcsP^HA^  =  image of fluorescence at 525 nm, IcsA  =  image of fluorescence at 625 nm, Merge  =  overlay of IcsP^HA^ and IcsA images. doi:10.1371/journal.pone.0070508.g005
