# Supplementary figures and images for: LPS Unmasking of Shigella flexneri Reveals Preferential Localisation of Tagged Outer Membrane Protease IcsP to Septa and New Poles
Source: PLoS One. 2013 Jul 25;8(7):e70508. doi: 10.1371/journal.pone.0070508 (PMC3723647; doi:10.1371/journal.pone.0070508)

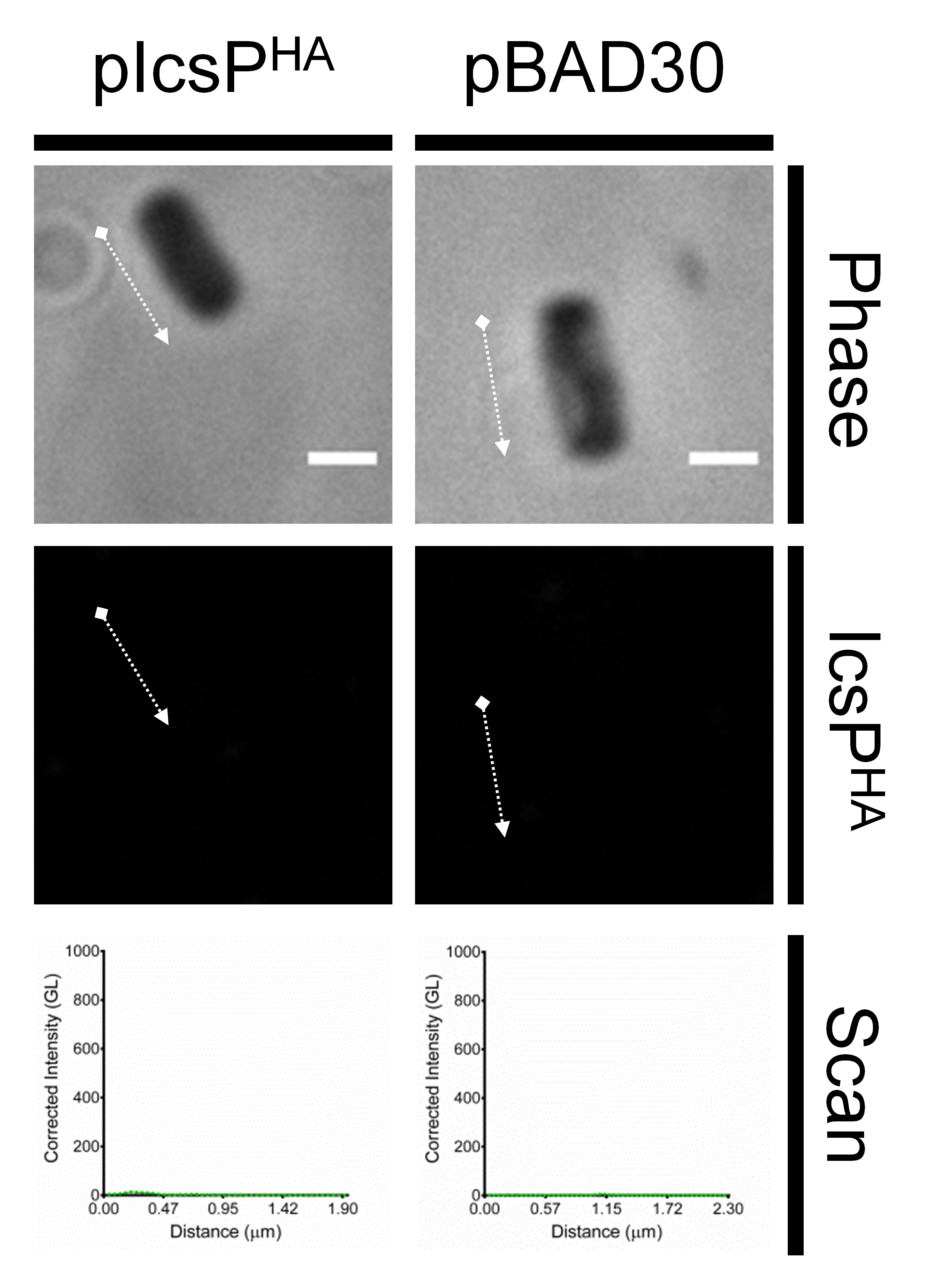

Supplement: Figure S1 — Inability to detect IcsPHA on the cell surface of 2457T icsP- . Smooth LPS 2457T icsP- strains expressing pIcsPHA (left column) or pBAD30 (right column) were subcultured in LB for 1.5 h to an OD600 reading of ∼0.4. Cultures were then washed 3 times in LB, induced with 0.03% (w/v) arabinose for 1 h, fixed, and subjected to QD IF using antibodies for HA epitope and IcsA. Representative bacteria are shown. Scan = Single line-scans measuring the intensity of IcsPHA detected along the surface of the bacterium, Bars = 1 µm, Arrows = direction of line-scan, GL = Grey level, Phase = phase contrast image, IcsPHA = image of fluorescence at 525 nm. (TIF) [file pone.0070508.s001.tif]

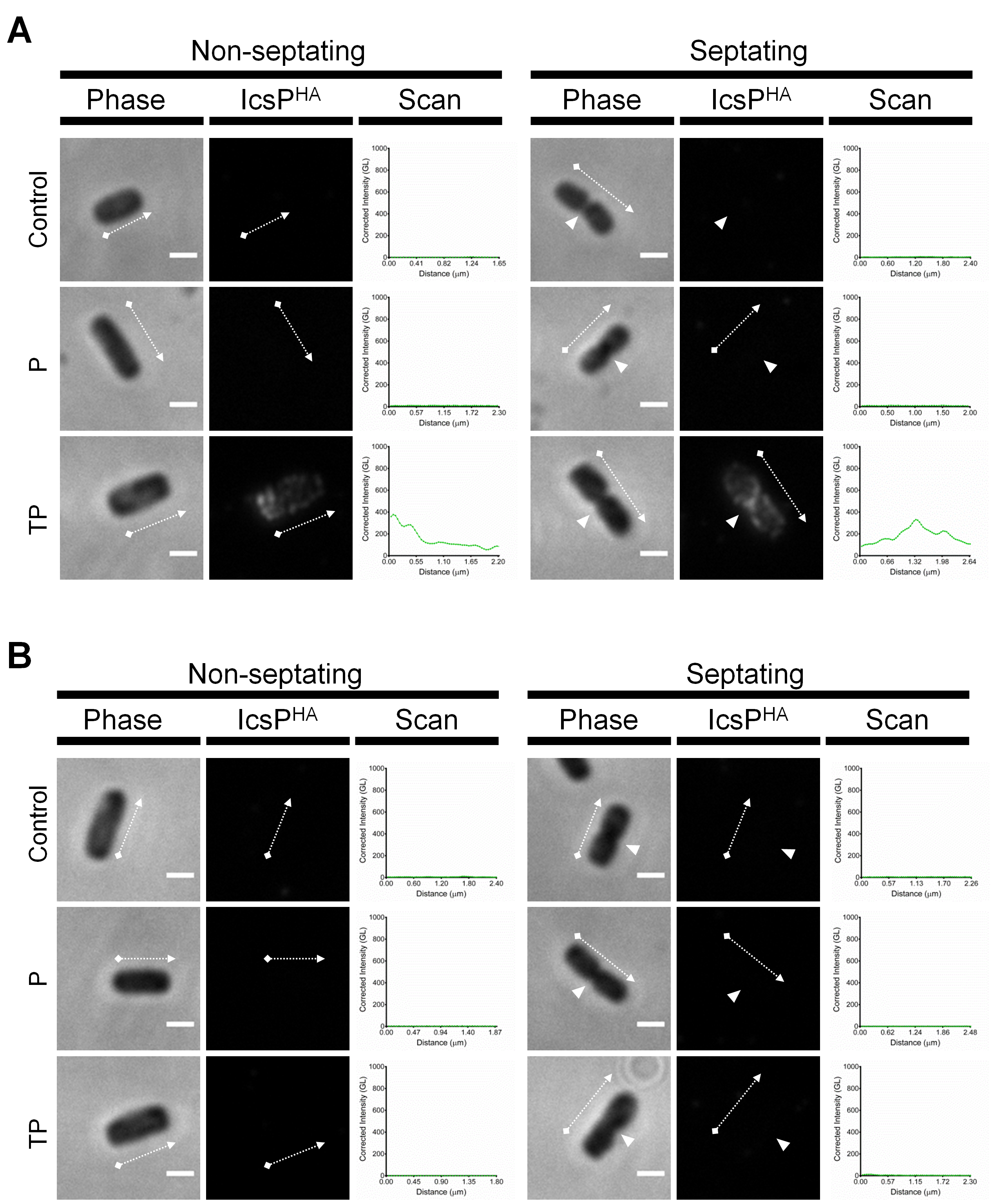

Supplement: Figure S2 — Single IcsPHA staining of LPS-depleted 2457T icsP- . Smooth LPS 2457T icsP- strains harbouring (A) pIcsPHA or (B) pBAD30 were subcultured in LB broth for 1.5 h to an OD600 reading of ∼0.4, washed 3 times in LB, and then further cultured for 2 h in either: the absence of TP, in the presence of PMBN only, or in the presence of TP. Arabinose was included in the final hour of treatment at a concentration of 0.03% (w/v). Samples were then fixed and subjected to QD IF using antibodies to HA epitope. Representative non-septating and septating cells are shown for each treatment group. Scan = Single line-scans measuring the fluorescence intensity of IcsPHA detected along the surface of the bacterium, Bars = 1 µm, Arrows = direction of line-scan, Arrow heads = septa, Control = grown in absence of both tunicamycin and PMBN, P = PMBN, TP = tunicamycin/PMBN, GL = Grey level, Phase = phase contrast image, IcsPHA = image of fluorescence at 525 nm (TIF) [file pone.0070508.s002.tif]

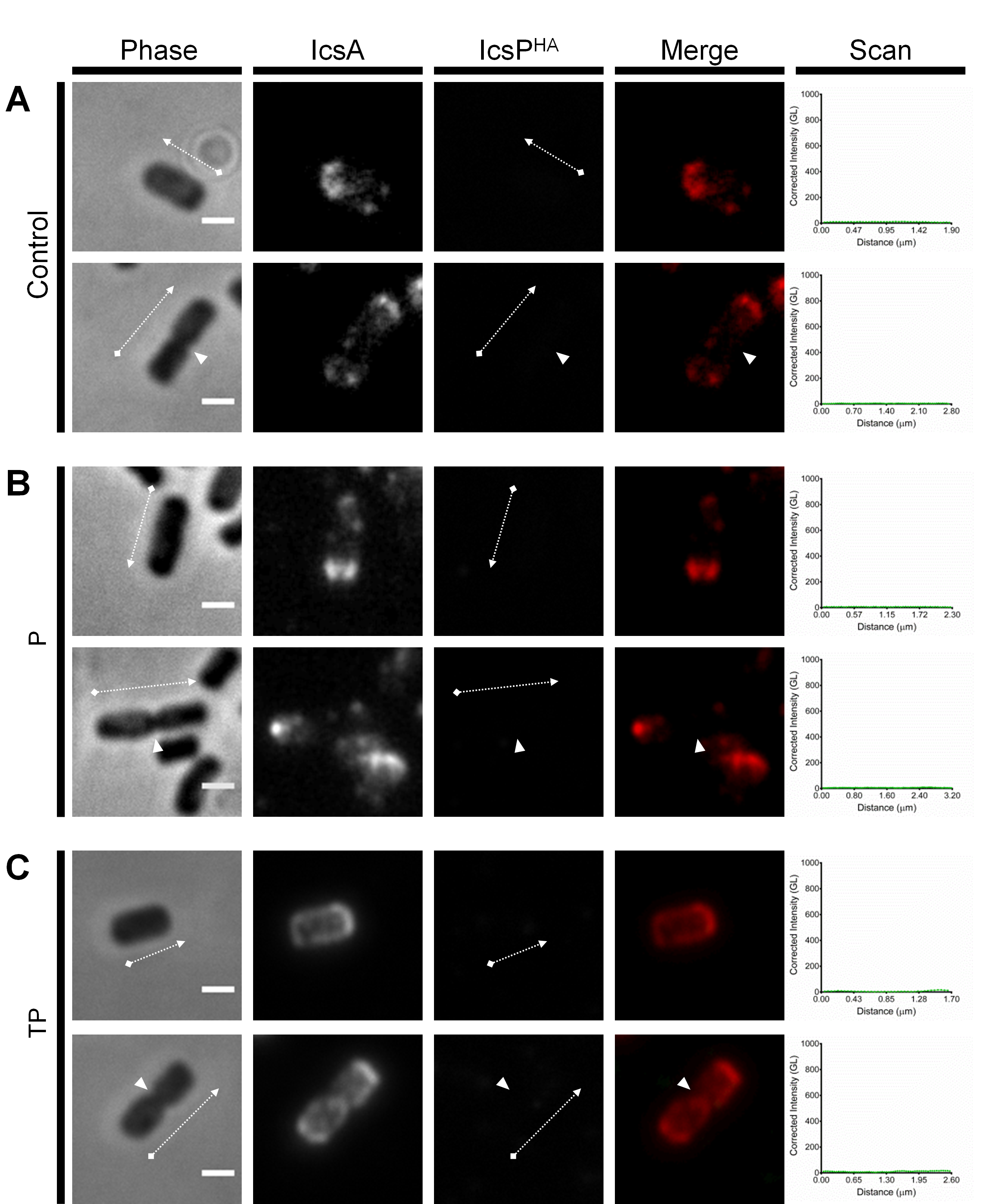

Supplement: Figure S3 — Double stained IF of LPS depleted 2457T icsP- [pBAD30]. Smooth LPS 2457T icsP- strains harbouring pBAD30 were subcultured in LB broth for 1.5 h to an OD600 reading of ∼0.4, washed 3 times in LB, and then further cultured for 2 h; (A) in the absence of TP, (B) in the presence of PMBN only, or (C) in the presence of TP. Arabinose was included in the final hour of treatment at a concentration of 0.03% (w/v). Samples were then fixed and subjected to QD IF using antibodies to HA epitope and IcsA. Non-septating and septating cells (upper and lower rows respectively) are shown for each treatment group. Representative bacteria are shown. Scan = Single line-scans measuring the fluorescence intensity of IcsPHA detected along the surface of the bacterium, Bars = 1 µm, Arrows = direction of line-scan, Arrow heads = septa, Control = grown in absence of both tunicamycin and PMBN, P = PMBN, TP = tunicamycin/PMBN, GL = Grey level, Phase = phase contrast image, IcsPHA = image of fluorescence at 525 nm, IcsA = image of fluorescence at 625 nm, Merge = overlay of IcsPHA and IcsA images. (TIF) [file pone.0070508.s003.tif]

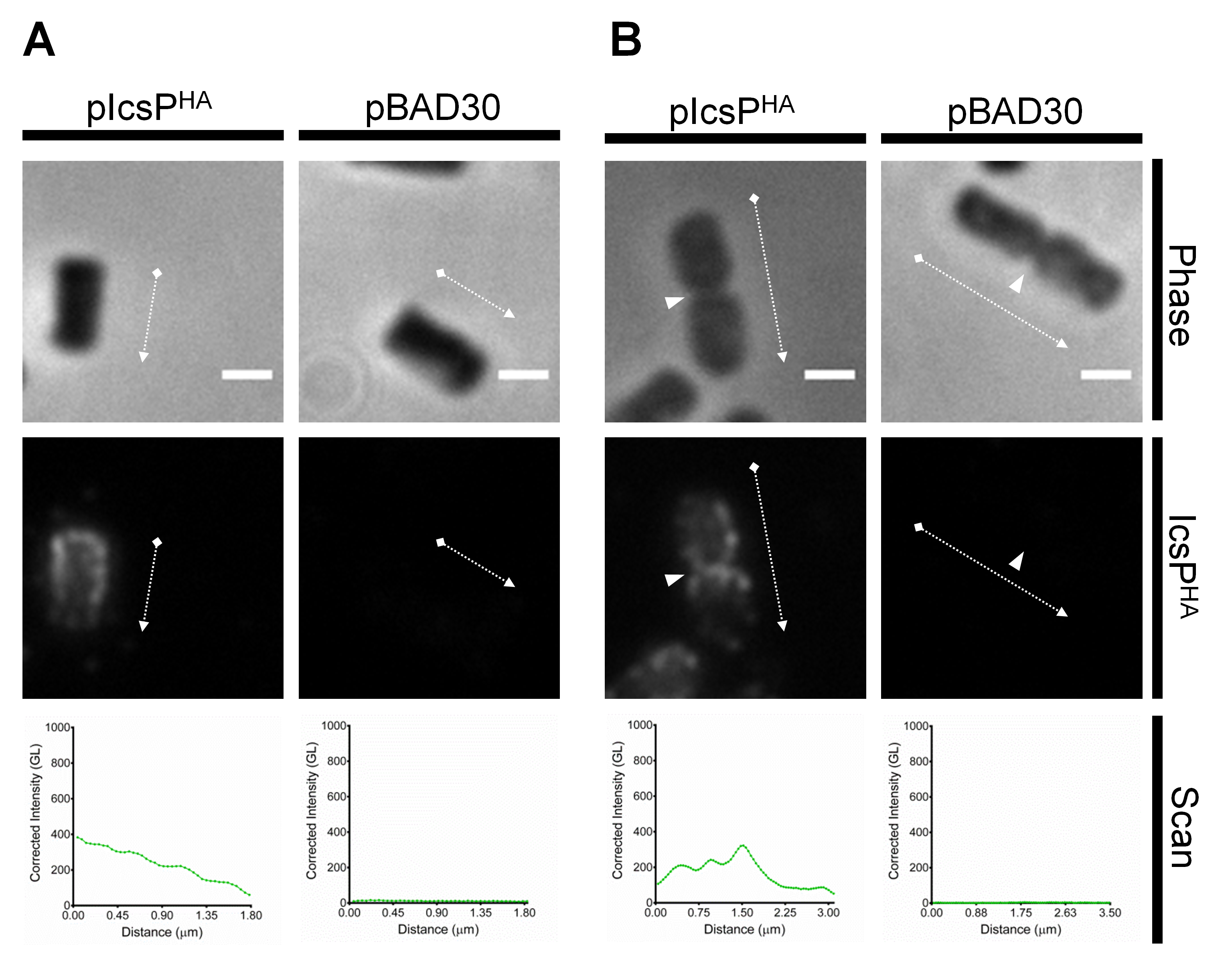

Supplement: Figure S4 — Single IcsPHA staining of 2457T icsP−/rmlD- . Rough LPS 2457T icsP−/rmlD- strains harbouring pIcsPHA (left columns) or pBAD30 (right columns) were subcultured LB broth for 1.5 h to an OD600 reading of ∼0.4. Cultures were then washed 3 times in LB, induced with 0.03% (w/v) arabinose for 1 h, fixed, and subjected to QD IF using antibodies for HA epitope. Non-septating and septating life stages are shown in A and B respectively. Representative bacteria are shown. Scan = Single line-scans measuring the fluorescence intensity of IcsPHA detected along the surface of the bacterium, Bars = 1 µm, Arrows = direction of line-scan, Arrow heads = septa, GL = Grey level, Phase = phase contrast image, IcsPHA = image of fluorescence at 525 nm. (TIF) [file pone.0070508.s004.tif]
